# SAL1‐PAP retrograde signaling orchestrates photosynthetic and extracellular reactive oxygen species for stress responses

**DOI:** 10.1111/tpj.70271

**Published:** 2025-06-12

**Authors:** Estee E. Tee, Stephen J. Fairweather, Hanh M. Vo, Chenchen Zhao, Andrew Breakspear, Sachie Kimura, Melanie Carmody, Michael Wrzaczek, Stefan Bröer, Christine Faulkner, Jaakko Kangasjärvi, Zhong‐Hua Chen, Barry J. Pogson, Kai Xun Chan

**Affiliations:** ^1^ Research School of Biology Australian National University 2601 Canberra Australia; ^2^ School of Science Western Sydney University 2753 Sydney Australia; ^3^ Cell and Developmental Biology John Innes Centre NR4 7UH Norwich UK; ^4^ Organismal and Evolutionary Biology, Viiki Plant Science Centre University of Helsinki 00014 Helsinki Finland; ^5^ Present address: Cell and Developmental Biology John Innes Centre NR4 7UH Norwich UK; ^6^ Present address: School of Natural Sciences University of Tasmania 7001 Hobart Australia; ^7^ Present address: Tasmanian Institute of Agriculture University of Tasmania 7250 Launceston Australia; ^8^ Present address: NAPIGEN, Inc. 19803 Delaware USA; ^9^ Present address: Synthetic Biology Future Science Platform Commonwealth Scientific and Industrial Research Organisation 2601 Canberra Australia; ^10^ Present address: Institute of Plant Molecular Biology Czech Academy of Sciences 370 05 České Budějovice Czech Republic; ^11^ Present address: Faculty of Science, Department of Experimental Plant Biology University of South Bohemia 370 05 České Budějovice Czech Republic

**Keywords:** chloroplast, ROS, retrograde signaling, PAP, SAL1, RESPIRATORY BURST OXIDASE HOMOLOG, calcium‐dependent protein kinase, 3′‐phosphoadenosine 5′‐phosphate (PAP)

## Abstract

Cellular responses to abiotic stress involve multiple signals such as reactive oxygen species (ROS), Ca^2+^, abscisic acid (ABA), and chloroplast‐to‐nucleus retrograde signals such as 3′‐phosphoadenosine 5′‐phosphate (PAP). The mechanism(s) by which these messengers intersect for cell regulation remain enigmatic, as do the roles of retrograde signals in specialized cells. Here we demonstrate a mechanistic link enabling ABA and PAP to coordinate chloroplast and plasma membrane ROS production. Contrary to its role in upregulating processes leading to quenching of ROS in foliar tissue, we show that in guard cells, PAP induces chloroplast ROS accumulation via photosynthetic electron transport and apoplast ROS via the RESPIRATORY BURST OXIDASE HOMOLOG (RBOH) proteins. Both subcellular ROS sources are necessary for stress hormone ABA‐mediated stomatal closure, as well as PAP‐mediated stomatal closure. However, PAP signaling diverges from ABA by activating RBOHD instead of RBOHF. Three calcium‐dependent protein kinases (CPKs) transcriptionally induced by PAP, namely CPK13, CPK32, and CPK34, concurrently activate RBOHD and the slow anion channel SLAC1 by phosphorylating two SLAC1 serine (S) residues, including S120, which is also targeted by the ABA signaling kinase OPEN STOMATA 1 (OST1). Consequently, overexpression of the PAP‐induced CPKs rescues stomatal closure in *ost1*. Our data identify chloroplast retrograde signals as critical nodes in cellular stress response networks of guard cells.

## Significance Statement

Chloroplast‐to‐nucleus retrograde signals intersect with multiple signaling pathways for cell regulation during abiotic stress but potentially function differently depending on the specialized cell. In contrast to previous studies showing the upregulation of ROS quenching processes by the retrograde signal 3′‐phosphoadenosine 5′‐phosphate (PAP) in foliar tissue, here we show that PAP induces ROS accumulation for stomatal closure, demonstrating how chloroplast signals intersect with subcellular ROS networks for cellular stress responses.

## INTRODUCTION

Plant responses to abiotic and biotic stresses involve a suite of coordinated cellular signaling cascades. These include retrograde signals, defined as signals from an organelle (i.e., the chloroplast or mitochondria) that regulate nuclear gene expression (Chan, Phua, et al., 2016; Cutler et al., 2010; Waszczak et al., 2018) and interact with multiple messenger molecules, including reactive oxygen species (ROS), calcium ions (Ca^2+^), and phytohormones such as abscisic acid (ABA). While each of the aforementioned messengers has been extensively studied, their spatio‐temporal intersections remain enigmatic. For example, both apoplastic ROS and organelle‐to‐nucleus retrograde signals occur concurrently, yet it is difficult to elucidate whether they affect one another and how they are both coordinated in specialized cells.

ROS are crucial signaling molecules produced in diverse subcellular compartments including chloroplasts and the apoplast. Several types of ROS exist in plant cells, and distinct ROS types play different signaling roles (Chan, Phua, et al., 2016; Cutler et al., 2010; Waszczak et al., 2018), but herein we refer to chloroplast and apoplast “ROS” is an encompassing term for three ROS types formed via similar mechanisms at these subcellular locations: superoxide ($O_{2}^{-}$), its dismutation product hydrogen peroxide (H_2_O_2_), and hydroxyl radical (OH^·^); with H_2_O_2_ being the most stable (Waszczak et al., 2018). Such ROS generated from photosystem I (PSI) in chloroplasts participate in chloroplast‐to‐nucleus retrograde signaling, both by triggering production of retrograde signals via H_2_O_2_‐responsive redox regulation (Chan, Mabbitt, et al., 2016) and by itself acting as a retrograde signal (Exposito‐Rodriguez et al., 2017). Perturbations to chloroplast ROS levels, either by silencing the chloroplastic H_2_O_2_ scavenging enzyme, thylakoid membrane‐bound ascorbate peroxidase (tAPX), or from the result of high light stress, which increases chloroplastic H_2_O_2_, lead to the up‐regulation of multiple stress‐associated genes, including pathogen‐responsive genes in photosynthetic tissues (Maruta et al., 2012). However, cellular targets of chloroplast‐sourced H_2_O_2_ that result in stress‐related functions are still largely understudied.

Leaf chloroplast PSI ROS can trigger other chloroplast‐to‐nucleus retrograde signaling pathways such as methylerythritol cyclodiphosphate (MEcPP) and SAL1‐PAP (3′‐phosphoadenosine 5′‐phosphate), the latter of which functions in drought and high light stress (Estavillo et al., 2011; Xiao et al., 2012). These abiotic stresses promote chloroplast PSI ROS and redox poise, which inhibit plastidial SAL1 activity (Chan, Mabbitt, et al., 2016), thus allowing accumulation of its catabolic substrate PAP in the cell. In the nucleus, PAP inhibits 5′ to 3′ exoribonucleases to alter RNA polymerase II function. This leads to read‐through accumulation of downstream genes in tandem repeats, resulting in accumulation of stress‐responsive transcripts including H_2_O_2_ scavengers such as *Ascorbate Peroxidase 2* (*APX2*) (Crisp et al., 2017, 2018). Consequently, *sal1* mutants with constitutively over‐accumulated PAP show lower foliar ROS levels, particularly in the vascular bundle (Estavillo et al., 2011).

In contrast to chloroplast PSI ROS, apoplastic superoxide and H_2_O_2_ are well known to act as secondary messengers in dynamic signaling cascades. One such well‐characterized pathway that utilizes apoplastic and cellular ROS as a secondary messenger is abscisic acid (ABA)‐mediated stomatal closure. In response to an accumulation of ABA under stress conditions such as drought, a simplified guard cell process occurs as follows: ABA forms a complex with PYRABACTIN RESISTANCE 1/PYR1‐LIKE/REGULATORY COMPONENTS OF ABA RECEPTORS, and ABSCISIC ACID‐INSENSITIVE 1 (ABI1) PP2C phosphatase then binds to the complex (Ma et al., 2009; Park et al., 2009). This removes the inhibition of SUCROSE NON‐FERMENTING‐1 RELATED PROTEIN KINASE (SnRK) 2.6 (SnRK 2.6)/SRK2E/OPEN STOMATA 1 (OST1), which can auto‐phosphorylate (Belin et al., 2006; Yoshida et al., 2006). OST1 interacts with RESPIRATORY BURST OXIDASE HOMOLOG F (RBOHF) and RBOHD, leading to RBOH‐mediated ROS (superoxide and H_2_O_2_) production (Acharya et al., 2013; Sirichandra et al., 2009). OST1 also directly regulates the activity of multiple ion channels by phosphorylation, including the activation of SLOW ANION CHANNEL 1 (SLAC1) and rapid type anion channel ALMT12 (Acharya et al., 2013; Geiger et al., 2009; Imes et al., 2013), and inhibiting inward rectifying POTASSIUM CHANNEL (KAT1) (Sato et al., 2009). While OST1 has the capacity to directly act on the channels, both ROS and cytosolic Ca^2+^ are required for stomatal closure. H_2_O_2_ activates Ca^2+^ channels to induce the influx of Ca^2+^ and the release of Ca^2+^ from vacuoles and other organelles to the cytosol (Pei et al., 2000; Wang et al., 2013; Wu et al., 2020), and other important proteins such as aquaporins modulate H_2_O_2_ levels in response to ABA (Rodrigues et al., 2017). As illustrated by Rodrigues et al. (2017), H_2_O_2_ can act as a central hub for stomatal signaling, but different components are utilized depending on the specific stimulus.

In guard cells, OST1 activation of RBOHF is thought to be the primary pathway for apoplastic ROS production (Sirichandra et al., 2009; Wu et al., 2020). ROS production via RBOHs is regulated by a suite of different kinases at the whole leaf, tissue, cellular, and organellar levels. The Ca^2+^ sensor CALCINEURIN B‐LIKE proteins (CBL1; CBL9) interact with CBL‐INTERACTING PROTEIN KINASE (CIPK11; CIPK26) to activate both RBOHF and RBOHD (Drerup et al., 2013; Han et al., 2019; Köster et al., 2025). However, RBOHD is thought to be the primary apoplastic ROS generator in response to biotic stimuli and mechanical wounding (Morales et al., 2016; Torres et al., 2002). Regulators of RBOHD activity include several immunity‐associated protein kinases (Lee et al., 2020; Li et al., 2014; Zhang et al., 2018) and calcium protein kinases (CPKs). CPK1, CPK2, CPK4, CPK5, CPK6, CPK11, and CPK16 can interact with either or both RBOHD and RBOHF (Dubiella et al., 2013; Gao et al., 2013; Yu et al., 2024).

The SAL1 protein is expressed in vascular bundles (Estavillo et al., 2011) as well as mesophyll and guard cells (Wang et al., 2023), suggesting the SAL1‐PAP pathway can function in multiple leaf cell types. Indeed, we previously found the SAL1‐PAP pathway functions in parallel to the canonical ABA signaling (RCAR/PYL‐OST1‐PP2C) pathway to induce stomatal closure, at least in part via transcriptional upregulation of CPKs, which converge upon and activate OST1 target proteins such as the SLAC1 anion channel (Pornsiriwong et al., 2017). Intriguingly, PAP appears to have contrasting effects on ROS in different cell types. While constitutive PAP accumulation leads to decreased ROS in vascular bundles (Estavillo et al., 2011), exogenous PAP application induces ROS in guard cells (Pornsiriwong et al., 2017). Constitutively accumulated PAP also rescues ABA‐induced ROS burst in *ost1 sal1* guard cells (Pornsiriwong et al., 2017). However, the mechanism was not investigated beyond establishing that most downstream canonical ABA‐regulated processes, such as ion fluxes, were activated by PAP in *ost1* mutants (Pornsiriwong et al., 2017). Given that stomatal guard cells have unique chloroplasts that play essential roles in sensing stimuli and activation of S‐type anion channels (Negi et al., 2018), this places the chloroplastic SAL1‐PAP pathway as a potential example of retrograde signaling in guard cells.

The links between PAP‐mediated chloroplast signaling, CPK activation, and the production of apoplastic ROS in guard cells remain elusive, if not illogical from a spatial context. Further, signaling roles for chloroplastic and apoplastic ROS have conventionally been considered separately; thus, till now, whether and how they intersect has remained unclear. Despite the parallels in chloroplast‐sourced retrograde ROS signaling and apoplast‐sourced secondary messenger ROS signaling, there is a distinct lack of knowledge on how these two processes are intertwined in cellular stress signaling. A more fundamental question is if retrograde signaling is an important cellular mechanism from an evolutionary perspective, how does it intersect with other stress signaling pathways?

To define how PAP, and consequently chloroplastic signals, can intersect with apoplastic ROS signaling, we use the guard cell as a model to study the specializations of different signaling pathways. This includes considerations of the sites of ROS accumulation, phosphorylation targets of CPKs, and the extent to which ABA‐mediated and PAP‐mediated signaling require chloroplast functionality as well as retrograde signaling for stomatal closure.

## RESULTS

### Subcellular guard cell ROS compartmentalization is mediated by chloroplast retrograde signaling

**Figure 1:**
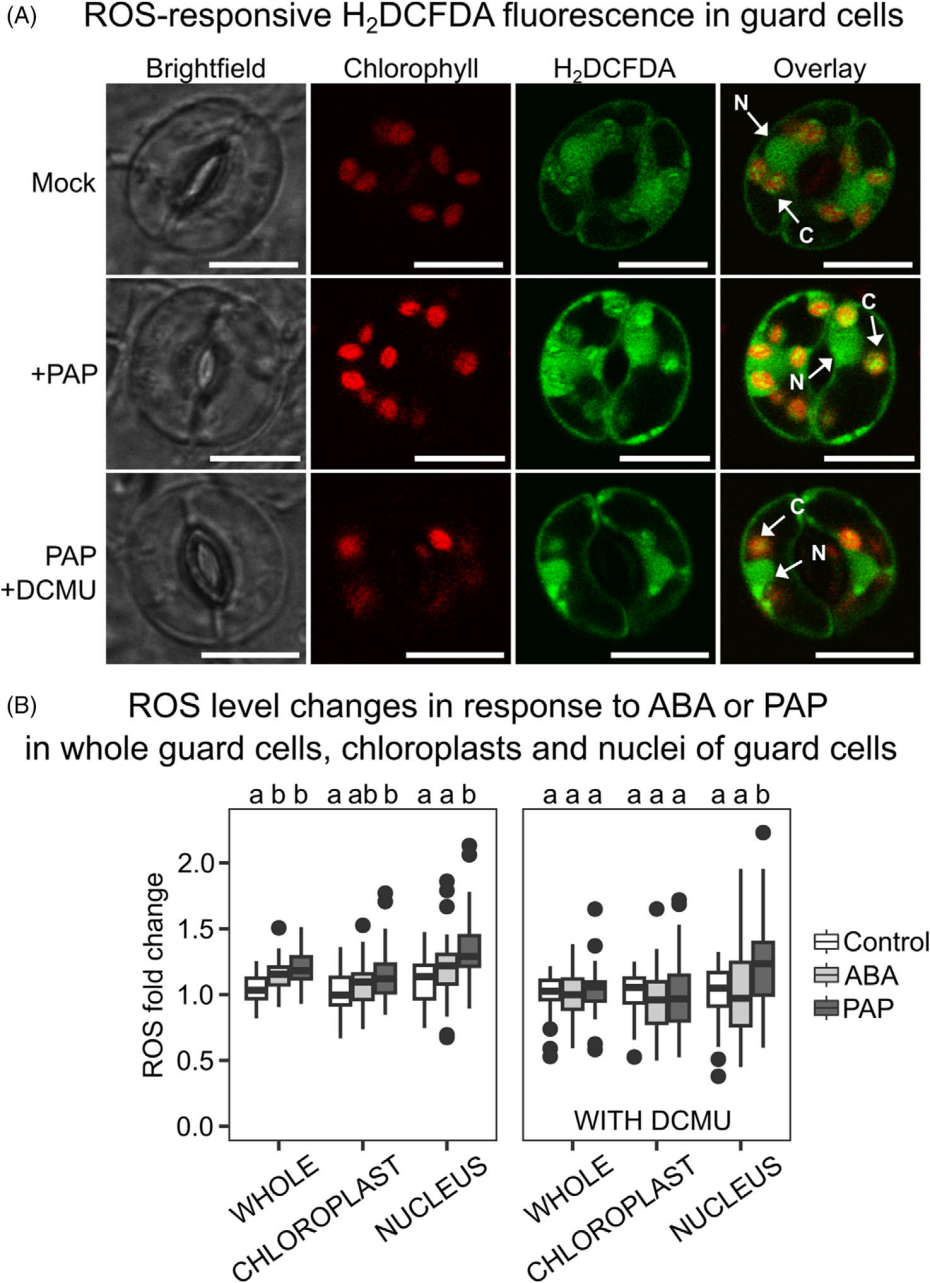
PAP induces ROS in the chloroplast and nucleus. (A) Localization of ROS within guard cells after 10 min' treatment with mock, PAP, or DCMU+PAP, with arrows indicating ROS at chloroplasts (C) and the nucleus (N). Scale bars = 10 μM. (B) Relative change in fluorescence (compared to before treatment) in different parts of the stomata after 10 min of treatment (100 μM ABA or 100 μM PAP), with or without 10 μM DCMU (labeled WITH DCMU). Fluorescence is an indication of ROS levels as per the ROS dye H_2_DCFDA. Each condition contains a minimum of three wild‐type plants with *n* ≥ 43 stomata per treatment combination. In the mock pre‐treatment (i.e., without DCMU), “Treatment” and “Part” significantly impacted fluorescence with no significant interaction between the two (ANOVA; *F* = 41.6, df = 2, *P* < 0.001; *F* = 28.4, df = 2, *P* < 0.001; *F* = 1.6, df = 4, *P* = 0.18). In the DCMU pre‐treatment, “Treatment” and “Part” significantly impacted fluorescence with a significant interaction between the two (ANOVA; *F* = 5.7, df = 2, *P* < 0.01; *F* = 5.8, df = 2, *P* < 0.01; *F* = 3.3, df = 4, *P* < 0.05). Significant differences relative to the respective control are denoted by a and b.

We have previously shown that ROS signaling was induced by PAP in guard cells. To determine the subcellular source and functions of PAP‐induced ROS, we examined the spatial distribution of ROS in response to exogenous PAP application using H_2_DCFDA, which acts as a general ROS marker by reacting with H_2_O_2_ and other radicals such as OH^·^ (Akter et al., 2021). H_2_DCFDA has been used to visualize ROS accumulation in whole leaves and subcellular compartments of guard cells (Postiglione & Muday, 2023; Watkins et al., 2017; Zandalinas et al., 2020). We observed that PAP and ABA elevated ROS in multiple guard cell subcellular compartments (Figure 1). Quantification of ROS‐responsive H_2_DCFDA fluorescence showed PAP significantly increased ROS in guard cell nuclei and chloroplasts to a higher extent than ABA (Figure 1B; no pre‐treatment). We verified that PAP induces an increase in ROS levels in the chloroplasts and nucleus of guard cells by respective overlay of H_2_DCFDA fluorescence with that of chlorophyll (Figure 1A) and two separate nuclear markers, Hoescht 33342 (Figure S1) and DAPI (Figure S2). We also visualized ROS accumulation in PAP‐treated guard cells using the H_2_O_2_‐specific fluorescent dye Peroxy Orange 1 (PO1) and found comparable PAP‐induced fluorescence increases in the guard cell between H_2_DCFDA and PO1 (Figure 1A; Figure S3).

PAP is reported to lower, not raise, chloroplast ROS in foliar tissues due to its retrograde action of upregulating ROS detoxification processes, such as *APX2* (Estavillo et al., 2011). Therefore, we tested the possibility that PAP might affect ROS differently in vascular bundles compared to guard cells. We confirmed that constitutive PAP accumulation in the *sal1* mutant suppresses vascular bundle ROS under both control and 1‐h high light stress conditions compared to wild type (Figure S4A). In contrast, PAP‐accumulating *sal1* guard cells showed significantly higher ROS‐responsive H_2_DCFDA fluorescence after 15 min and 1 h of ABA treatment compared to wild type (Figure S4B). This enhancement of ROS levels in *sal1* guard cells is consistent with the effect of exogenous PAP on wild‐type guard cells (Figure 1), suggesting that PAP indeed induces ROS accumulation in guard cells.

We further investigated the interaction between PAP and chloroplastic ROS using biochemical and genetic tools. The chloroplast photosynthetic electron transport chain is a major source of ROS in chloroplasts (Waszczak et al., 2018). Therefore, we attempted to attenuate chloroplastic ROS production using different concentrations of the photosynthesis inhibitor 3‐(3‐4,‐dichlorophenyl)‐1,1‐dimethylurea (DCMU) on *Arabidopsis* epidermal peels. As expected, we observed an incremental reduction in the effective PSII quantum yield (Y(II)) of mesophyll tissue in response to the increasing concentrations of DCMU (Figure S5); whereas Y(II) in the guard cells was below the detection threshold. We used this decrease in Y(II) in the mesophyll to infer that Y(II) was similarly decreased in the chloroplasts of guard cells given the similarity of chloroplast photosynthesis in both cell types (Lawson et al., 2003, 2008).

**Figure 2:**
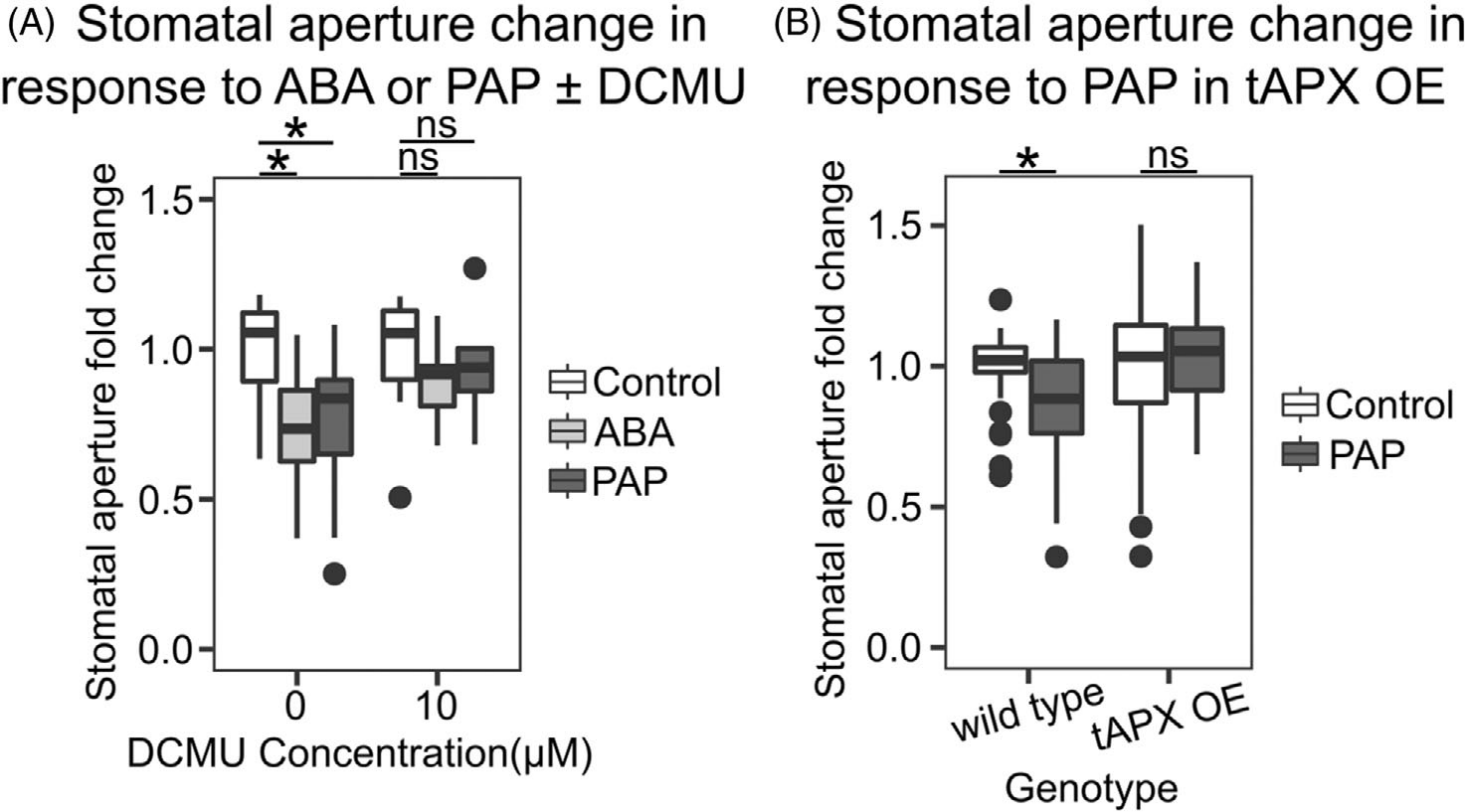
Chloroplastic ROS is required for PAP‐mediated stomatal closure. (A) Stomatal aperture in response to 10 min treatment of 100 μM ABA or 100 μM PAP with 0 or 10 μM DCMU, fold change relative to DCMU control (i.e., 0 DCMU or 10 μM DCMU). Each condition contains a minimum of three wild‐type plants with a minimum of *n* ≥ 27 stomata per treatment combination. “DCMU” and “Treatment” significantly impacted closure (ANOVA; *F* = 7.7, df = 1, *P* < 0.01; *F* = 4.2, df = 2, *P* < 0.05), with significant differences from respective control determined denoted by *P < 0.05. (B) Stomatal closure in response to 10 min treatment of 100 μM PAP in tAPX OE. Each condition contains a minimum of three biological replicates with a minimum of *n* ≥ 41 stomata per treatment combination. “Genotype” significantly impacted closure (ANOVA; *F* = 5.4, df = 1, *P* < 0.05), with a significant interaction between “Genotype” and “Treatment” (ANOVA; *F* = 4.9, df = 1, *P* < 0.05). Significant differences from the genotype control are denoted by *P < 0.05.

Inhibition of guard cell photosynthesis and chloroplastic ROS production at 10 μM DCMU decreased both ROS accumulation in guard cells and guard cell chloroplasts treated with ABA or PAP (Figure 1A). Interestingly, DCMU prevented ROS accumulation in the nuclei of guard cells treated with ABA but did not for PAP. The decreased ROS correlated with impaired ABA‐ or PAP‐induced stomatal closure when co‐treated with a range of DCMU concentrations (Figure 2A; Figure S6).

To further test if chloroplastic PSI ROS contributes to PAP‐mediated stomatal closure, we utilized a thylakoid ascorbate peroxidase (tAPX) over‐expressor (tAPX‐OE) line (Murgia et al., 2004). This transgenic line has high levels of tAPX activity for catalyzing the reduction of chloroplastic H_2_O_2_ to water. We hypothesized that increased scavenging of chloroplastic PSI ROS would prevent PAP‐mediated stomatal closure. Indeed, PAP‐mediated stomatal closure was inhibited in tAPX‐OE in comparison to wild type (Figure 2B). Our results indicate that, like ABA, PAP induces ROS production in guard cell chloroplasts, with the ROS capable of accumulating to high levels in the nucleus.

### Apoplastic ROS production by RBOHD is required for PAP‐mediated signaling processes

**Figure 3:**
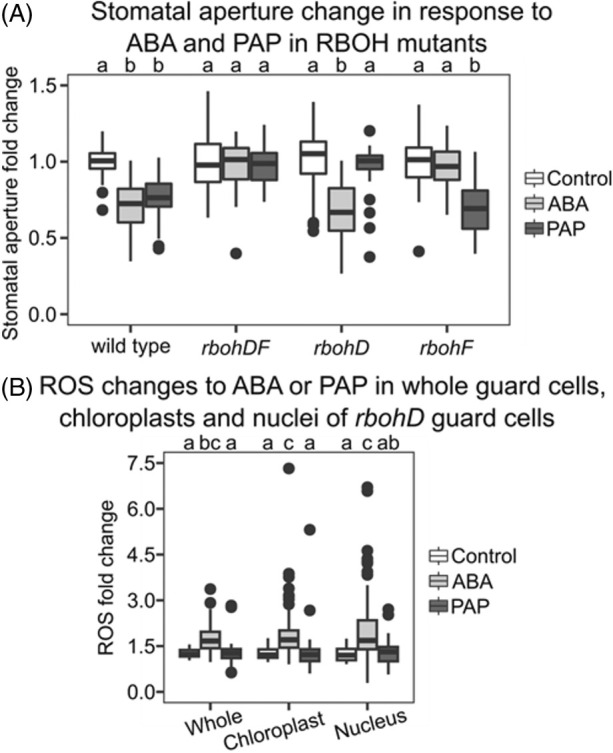
*rboh* mutants are differentially perturbed in ABA and PAP‐mediated stomatal closure. (A) Stomatal closure of *rboh* mutants in response to 10 min treatment with 100 μM ABA or 100 μM PAP. Each condition contains a minimum of three biological replicates with a minimum of *n* ≥ 34 stomata per treatment combination. “Genotype” and “Treatment” significantly impacted closure with an interaction between the two (ANOVA; *F* = 15.8, df = 3, *P* < 0.001; *F* = 38.6, df = 2, *P* < 0.001; *F* = 18.1, df = 6, *P* < 0.001). Significant differences to the respective genotype control are denoted by a or b. (B) Relative change in ROS‐responsive H_2_DCFDA fluorescence in whole guard cells, chloroplasts, and nuclei of *rbohD* guard cells after 10 min of 100 μM ABA or 100 μM PAP treatment. Each condition contains a minimum of three biological replicates with a minimum of *n* ≥ 36 stomata per treatment combination. “Treatment” significantly impacted fluorescence (ANOVA; *F* = 45.6, df = 2, *P* < 0.001) while “Cellular Component” and the interaction between the two did not (ANOVA; *F* = 2.1, *P* = 0.1; *F* = 1.5, *P* = 0.2). Significant differences are denoted by a, b, and c, *P* < 0.05.

While apoplastic ROS is required for stomatal closure mediated by ABA (Rodrigues et al., 2017), the role and genetic components for this process have not been identified for PAP‐mediated stomatal closure. We found that the double mutant *rbohDrbohF* (*rbohDF*) did not close stomata in response to PAP and had the expected impaired ABA‐mediated stomatal closure (Kwak et al., 2003) (Figure 3A). Interestingly, we found that the single knockout *rbohF* mutant was responsive to PAP but had completely impaired ABA‐mediated stomatal closure (i.e., no different from the control). In contrast, ABA induced stomatal closure in *rbohD*, but PAP could not. Given the findings that both ABA and PAP induce ROS in wild‐type guard cells and wild‐type guard cell chloroplasts (Figure 1B) we investigated *rbohD* responses and found that while ABA induced a significant increase in ROS of *rbohD* guard cells, PAP treatment on *rbohD* did not (Figure 3B). This was also the case when chloroplastic ROS was measured by quantifying H_2_DCFDA fluorescence specifically in areas overlapping with chlorophyll fluorescence (Figure 3B). These findings correlate with ABA‐mediated stomatal closure and PAP not inducing stomatal closure in *rbohD*. This indicates that ABA induces ROS via RBOHF, whereas PAP acts through RBOHD, which marks an important delineation between ABA‐ and PAP‐mediated stomatal closure.

The requirement of RBOHD for PAP‐induced chloroplastic ROS (Figure 3B) would suggest that the production of plastidial ROS is initiated by extracellular ROS in this signaling cascade. We then tested the reverse hypothesis, that is, apoplastic ROS production requires chloroplastic ROS. We utilized a modified form of H_2_DCFDA, Oxyburst Green H2HFF‐BSA, which is restricted to the apoplast and thus acts as a general apoplast ROS marker (Miller et al., 2010). PAP treatment induced an increase in apoplastic Oxyburst Green H2HFF‐BSA fluorescence in guard cells compared to mock treatment, as expected (Figure S7). Inhibition of chloroplast ROS production significantly decreased, but did not completely abolish, apoplastic Oxyburst Green H2HFF‐BSA fluorescence in guard cells co‐treated with PAP and DCMU (Figure S7). Taken together, our data suggest that the effect of PAP on subcellular ROS is multifaceted, with PAP‐induced ROS production in chloroplasts and the apoplast influencing each other.

### PAP‐induced CPKs activate RBOHD potentially as a means for apoplastic ROS production

We sought to identify the mechanism(s) by which PAP activates RBOHD‐mediated apoplastic ROS production. Induction of RBOH activity for ROS production requires N‐terminal phosphorylation activation by protein kinases such as OST1 and CPKs (Acharya et al., 2013; Dubiella et al., 2013; Lee et al., 2020; Li et al., 2014; Sirichandra et al., 2009; Zhang et al., 2018). We previously showed that upregulated PAP levels via *sal1* mutation could complement the loss of OST1 for stomatal regulation, including in ABA‐responsive ROS production, with PAP treatment also inducing ROS accumulation in *ost1*‐2 (Pornsiriwong et al., 2017). These results were further confirmed in whole plants exposed to ozone, which triggers a biphasic ROS accumulation in guard cells, with the induction first observed in chloroplasts. The *ost1*‐3 mutant has lower ROS production in response to ozone, attributed to its role in regulating apoplastic RBOH‐mediated ROS production, and is consequently sensitive to ozone treatment (Vahisalu et al., 2010). We measured cell death in Col‐0 (wild type), *ost1*‐3, *sal1*‐6, and *ost1*‐3 *sal1*‐6 in response to ozone. Ozone‐tolerant wild‐type and ozone‐sensitive *ost1*‐3 plants showed the expected phenotypes, with lesions formed in the midrib of all leaves of *ost1*‐3 (Figure S8A). Interestingly, fewer lesions developed on *ost1‐3* *sal1*‐6; and said lesions were primarily contained to the older leaves in the lower part of the rosette. In accordance with this, whole‐plant electrolyte measurements showed that the significantly greater extent of ozone‐induced ion leakage in *ost1*‐3 was decreased to wild‐type levels in *ost1*‐3 *sal1‐6* (Figure S8B). This suggests that the effect of PAP on apoplastic ROS signaling does not require OST1.

**Figure 4:**
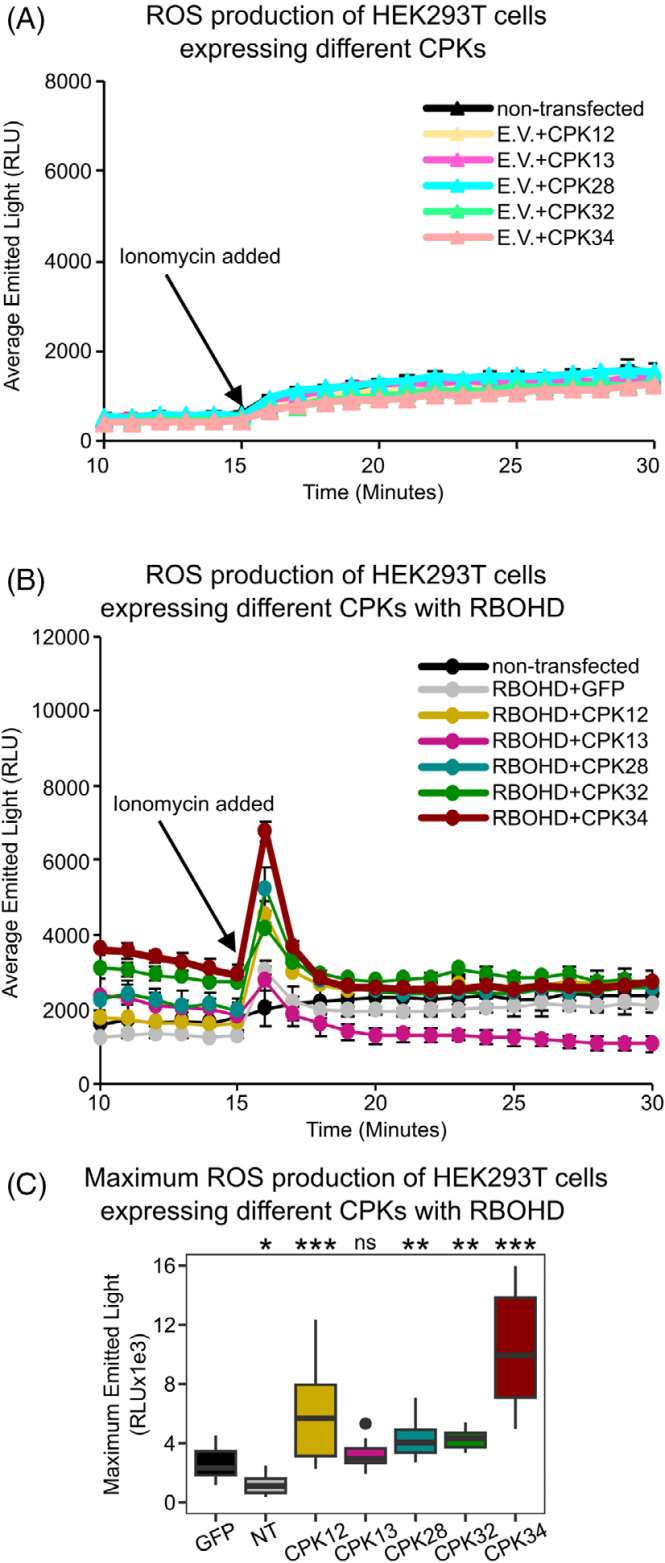
Different PAP‐induced CPKs enhance RBOHD‐mediated ROS production. HEK293T cells were expressing proteins of interest. After 15 min, 1 μM ionomycin was added to the medium. (A) and (B) ROS production of HEK293T cells transiently expressing CPKs with an empty vector (E.V.) or RBOHD. For (B), the experiment was repeated in at least four independent experiments with similar results. Values represent mean ± SEM. (C) Boxplot shows average max emitted light of non‐transfected control (NT) or RBOHD expressed with GFP or specified CPK; data pooled from separate experiments. GFP *n* = 24, NT *n* = 21, CPK12 *n* = 18, CPK13, 28, 32, and 34 *n* = 12. “Gene” expressed with RBOHD significantly impacted the max emitted light after ionomycin was added (ANOVA; *F* = 41.2, df = 6, *P* < 0.001), with significant differences from GFP denoted as * for *P* < 0.05, ** for *P* < 0.01, or *** for *P* < 0.001.

Given that CPKs are also activators of RBOHD (Dubiella et al., 2013), we investigated CPKs shown to be transcriptionally up‐regulated by PAP (Pornsiriwong et al., 2017) as potential enhancers of RBOHD‐mediated ROS production. To determine whether these candidate CPKs could enhance the ROS‐producing activity of RBOHD, we used heterologous expression in human embryonic kidney 293T (HEK293T) cells. These cells lack endogenous ROS production activity under experimental conditions employed herein (Bánfi et al., 2001) and are routinely used to investigate post‐translational regulation of RBOH activity (Chu et al., 2023; Kaya et al., 2019). First, we investigated whether the CPKs alone could produce ROS. Measurement of ROS by luminol‐amplified chemiluminescence showed that the single transfections of CPKs alone did not differ significantly from the non‐transfected control, indicating that the CPKs alone could not induce ROS production (Figure 4A).

HEK293T cells were then co‐transfected with *3FLAG‐RBOHD* and either *CPK‐3Myc* or *3Myc‐GFP*, which served as a protein expression control (Figure 4B,C). RBOHD protein levels were homogenous between co‐transfections, indicating that any differences in ROS production would be due to an additional protein partner (Figure S9). Upon stimulation with ionomycin, which supplies Ca^2+^ as a cofactor of RBOH proteins, HEK293T cells co‐expressing *3FLAG‐RBOHD* and the negative control *3Myc‐GFP*, which would not be expected to enhance RBOHD‐mediated ROS production, exhibited an increase in luminescence when compared to the non‐transfection negative control.

We then examined whether HEK293T cells co‐transfected with 3*FLAG‐RBOHD* and either CPK12, CPK13, CPK28, CPK32, or CPK34 (fused with *3Myc*) enhanced RBOHD‐mediated ROS production, that is, more than the *3Myc‐GFP* with *3FLAG‐RBOHD*, which serves as the baseline RBOHD‐mediated ROS production. CPK13 did not enhance ROS production when compared to the GFP control, despite CPK13 being expressed to higher levels than CPK28 or CPK32 in the HEK293T cells (Figure S9). In comparison, CPK12, CPK28, CPK32, and CPK34 enhanced the ionomycin‐stimulated RBOHD‐mediated ROS production by between 3‐ and 10‐fold, which was statistically significant compared to the GFP control. Overall, these results demonstrate that a specific subset of CPKs which are known to be transcriptionally up‐regulated by PAP can activate RBOHD for PAP‐mediated ROS functions.

### PAP‐induced CPKs activate RBOHD and SLAC1, thereby simultaneously impacting ROS and anion currents in stomatal closure

**Figure 5:**
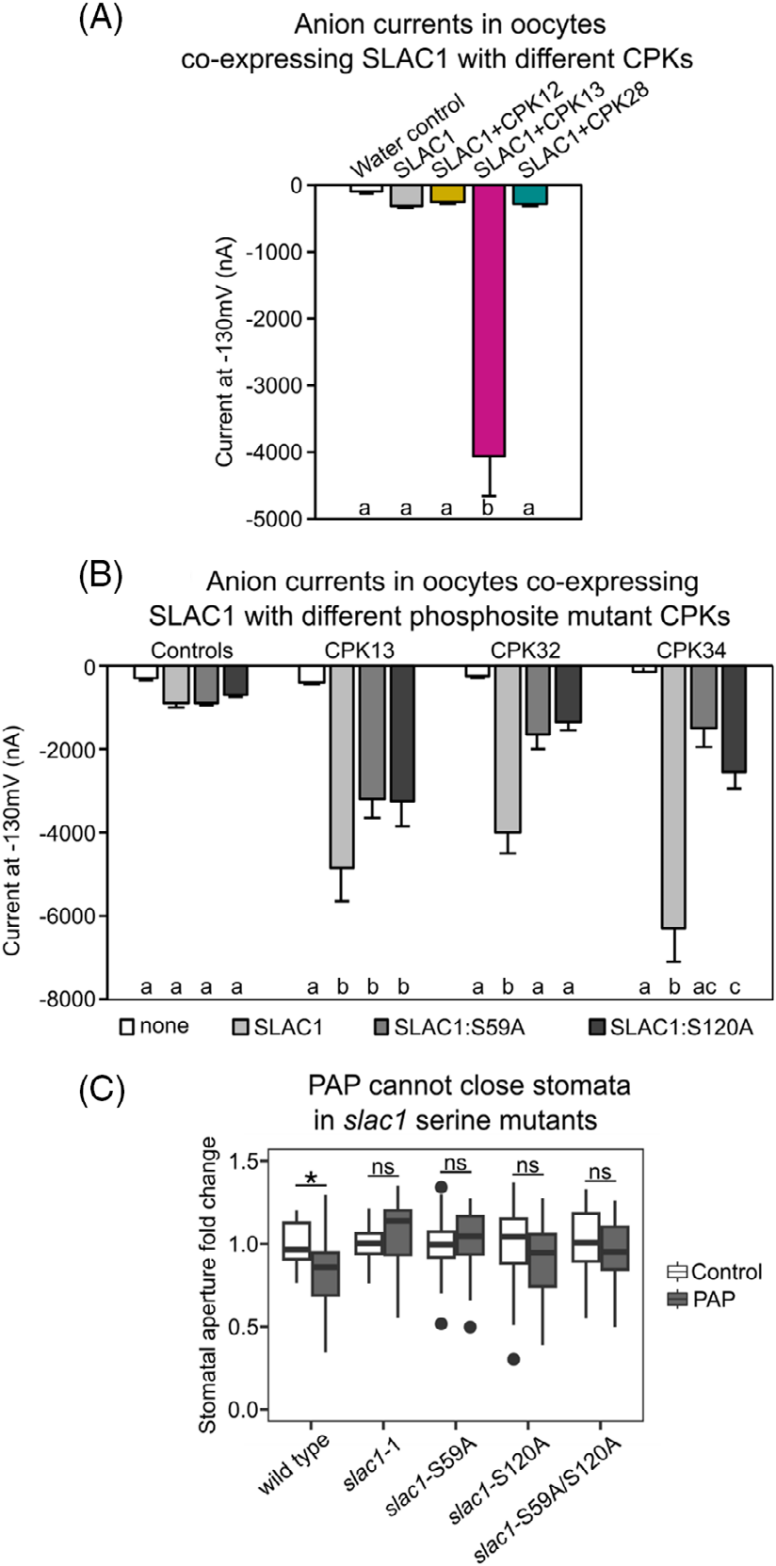
Different PAP‐induced CPKs enhance SLAC1 anion currents. (A) CPK12, CPK13, and CPK28 activation of the SLAC1 anion channel in oocytes. Values are the means of four to eight oocytes ± SEM. Anion channel activity is shown as steady‐state currents activated at −130 mV, with significant differences between injected combinations (ANOVA; *F* = 4.9, df = 4, *P* < 0.001), denoted by a and b. The experiment was repeated on positive candidates in two other batches of oocytes. See Figure S10 for the full trace. (B) Anion currents of mutated phosphosites in SLAC1 when co‐expressed with CPK13, CPK32, and CPK34 in oocytes. Values are from a minimum of five oocytes per combination ± SEM. Factors “Type of SLAC1 expressed” and “CPK” expressed significantly impacted the anion currents, with a significant interaction between the two (ANOVA; *F* = 70.8, df = 3, *P* < 0.001; *F* = 33.5, df = 3, *P* < 0.001; *F* = 16.8, df = 9, *P* < 0.001). Significant differences with a given CPK expressed, denoted by a, b, and c. (C) Stomatal aperture of *slac1* mutants in response to 100 μM PAP. Each has a minimum of three biological replicates with a minimum of *n* ≥ 31 stomata per genotype/treatment combination. Factor “Genotype” significantly impacted closure, factor “Treatment” did not, and there was a significant interaction between the two (ANOVA; *F* = 3.4, df = 4, *P* < 0.01; *F* = 3.4, df = 1, *P* = 0.07; *F* = 3.9, df = 4, *P* < 0.01). Significant difference in treatment within a genotype denoted by *P < 0.05.

The RBOHD‐activating kinases include CPK32 and CPK34. We have previously shown that these CPKs are capable of activating the slow anion channel SLAC1 (responsible for Cl^−^ and ${\mathrm{NO}}_{3}^{-}$ efflux from guard cells during stomatal closure) when co‐expressed in *Xenopus laevis* oocytes (Pornsiriwong et al., 2017). This raises the interesting question of which, among the repertoire of PAP‐upregulated CPKs, might target RBOHD and SLAC1 together or individually. We therefore tested CPK12, CPK13, and CPK28 for SLAC1 activation using the *Xenopus* heterologous expression system (Figure 5A; Figures S10 and S11). CPK12 could not activate SLAC1 in oocytes, consistent with findings by Brandt et al. (2015), nor could CPK28 elicit SLAC1 anion currents. However, CPK13 could elicit SLAC1 anion currents. Overall, different members of the PAP‐induced CPKs target RBOHD and SLAC1 either individually or collectively (Table S1).

OST1 and CPK6 kinases phosphorylate different serine sites on SLAC1 (Brandt et al., 2012, 2015; Geiger et al., 2009). Serine 120 (S120) is phosphorylated by OST1, while CPK6 phosphorylates S59 (Brandt et al., 2015). To define whether some of our identified CPK candidates target these phosphorylation sites, S59 and S120 of SLAC1 were individually or both mutated to alanine. SLAC1:S59A and SLAC1:S120A were individually co‐expressed in oocytes with CPK13, CPK32, and CPK34 (Figure 5B; Figure S11). Contrary to the published literature on OST1 and CPKs phosphorylating distinct sites on SLAC1, either one of the S59A or S120A mutations in SLAC1 on its own led to a reduction in anion currents for CPK32 and CPK34. When we mutated both serine sites (i.e., SLAC1‐S59A:S120A), this led to a further reduction in anion currents for CPK13 (Figure S11).

We investigated these findings *in planta* by testing whether different *Arabidopsis* SLAC1 mutants responded to PAP. First, we confirmed that, as expected, PAP and ABA did not close stomata of *slac1*‐4 lacking the SLAC1 protein (Figure S12). Next, we utilized *slac1*‐1 complemented with SLAC1 containing mutated serine sites of either S59A, S120A, or both S59A/S120A; in comparison to wild type, all mutants did not close stomata when treated with PAP (Figure 5C).

**Figure 6:**
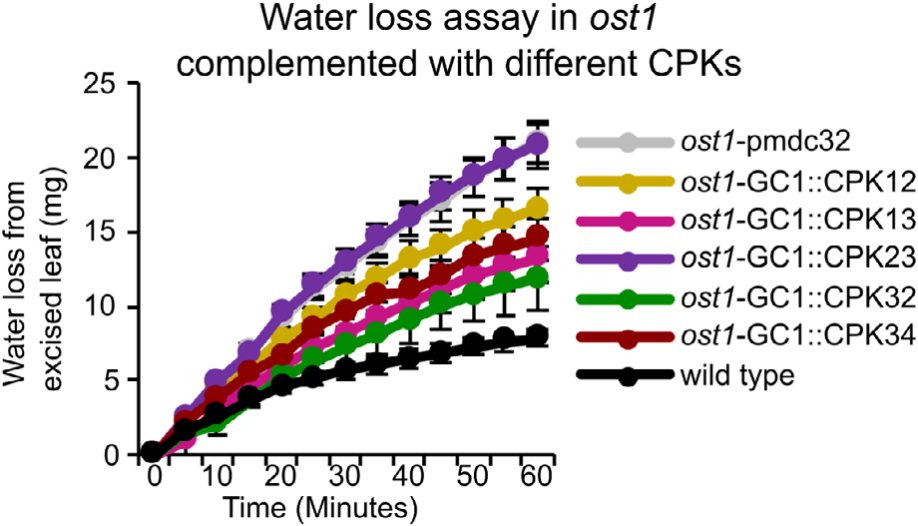
Different PAP‐induced CPKs can compensate for the loss of *OST1*. Water loss assay in excised leaves of *ost1* complemented with guard‐cell promoter‐expressed CPKs. Independent transgenic lines, where *n* = 8, 17, 14, 9, 13, 6, 14, and 11, respectively, for *ost1*‐pmdc32, *ost1*‐GC::CPK12, *ost1*‐GC::CPK13, *ost1*‐GC::CPK23, *ost1*‐GC::CPK32, *ost1*‐GC::CPK34, and wild type.

Considering these PAP‐related CPKs could activate both RBOHD and the anion channel SLAC1 individually, or both, thereby simultaneously impacting ROS and anion currents in stomatal closure, we wanted to determine whether they could compensate for the lack of OST1, an important kinase that activates SLAC1 and RBOHF (Acharya et al., 2013; Sirichandra et al., 2009). We transgenically overexpressed the different CPKs in the *ost1* background and performed a water‐loss assay on excised individual leaves of these transgenic lines. We included CPK23, a known activator of SLAC1 involved in the OST1‐dependent ABA signaling pathway (Geiger et al., 2010) but not transcriptionally PAP‐induced. Expressing CPK23 in *ost1* did not alter the water‐loss rate when compared to the empty‐vector *OST1* control (Figure 6). In contrast, the overexpression of PAP‐induced CPKs in *OST1* could reduce the water loss in comparison to empty‐vector *ost1* to varying degrees (Figure 6). This indicates that PAP‐induced CPKs, in particular CPK32 and CPK34, can, when expressed singularly, partially compensate for the lack of OST1 *in vivo* by modulating RBOHD and/or SLAC1 activity (Table S1).

## DISCUSSION

### PAP unexpectedly induces ROS in multiple subcellular compartments, including chloroplasts

We observed that the retrograde signal, PAP, identified in plants for its capacity to quench ROS in the vascular bundle (Estavillo et al., 2011), has a different role in guard cells where it functionally complements ABA signaling. Collectively, the pattern of PAP‐induced ROS accumulation in both chloroplasts and the nucleus of guard cells is very similar to that induced by ABA (Figure 1; Figure S3). This suggests that increases in chloroplast and nuclear ROS can potentially be utilized across different signaling cascades for stomatal regulation (Postiglione & Muday, 2023; Watkins et al., 2017). Our ABA results are consistent with recent reports of ABA inducing ROS accumulation in chloroplasts, nuclei, and mitochondria of *Arabidopsis* and tomato guard cells (Postiglione & Muday, 2023; Watkins et al., 2017). These authors found similar extents in ABA‐induced ROS‐triggered changes when measured using diverse tools including the chemical reporters H_2_DCFDA and PO1 (as performed in this study) or the pH‐insensitive genetically encoded H_2_O_2_ biosensor protein roGFP2‐Orp1 (Postiglione & Muday, 2023).

The inhibition of PAP‐induced stomatal closure by DCMU or tAPX over‐expression under low light (Figure 1; Figure S6) identifies PSI and the requirement for a functioning photosynthetic electron transport chain as likely origins of the chloroplastic ROS required for stomatal closure. Intriguingly, this PAP‐induced increase in ROS level driven by the light reactions of photosynthesis was not associated with any changes to the low light regime; that is, there was no requirement for co‐treatment with high light as would be typically expected for chloroplastic ROS production. It seems, even under low light, that chloroplastic PSI ROS and photosynthesis *per se* could play important roles in guard cell signaling, with PAP, ABA, and apoplastic ROS among the triggers for increases in photosynthetic‐generated ROS (Figure 1; Watkins et al., 2017; Postiglione & Muday, 2023). There could be a synergistic interaction between chloroplastic ROS and RBOH‐mediated ROS for stomatal closure, such as facilitating calcium release, thereby promoting activation of ion channels via CPKs (Demidchik & Shabala, 2018; Sierla et al., 2016). Consistent with this assumption, suppression of chloroplast ROS with DCMU decreased PAP‐induced apoplastic ROS (Figure S8).

It is unknown whether there is a direct transfer of H_2_O_2_ from chloroplasts to the nucleus in guard cells during stress signaling. There are at least two potential mechanisms for this process. First, close contact between chloroplasts and the nucleus has been proposed to enable chloroplast‐sourced H_2_O_2_ to bypass the cytosol and directly enter the nucleus of *Nicotiana benthamiana* abaxial epidermal cells (Exposito‐Rodriguez et al., 2017). Second, stroma‐filled tubular projections from plastids, termed stromules, can transfer H_2_O_2_ from chloroplasts to the nucleus during plant immune responses (Caplan et al., 2015). Stromule formation can be induced by ROS accumulation and ABA treatment, and chloroplasts with stromules have also been observed in guard cells (Brunkard et al., 2015). In our experiments (Figure 1A), some chloroplasts were in close contact with the nucleus, but we did not detect any stromules. Nevertheless, as stromules are also involved in enabling perinuclear clustering of chloroplasts (Jung et al., 2024; Mullineaux et al., 2020), it may be that the chloroplast‐nuclear association in our observations was enabled at least in part by stromules. A detailed investigation using sensitive H_2_O_2_ probes such as Hyper7 (Ugalde et al., 2021) and genetic backgrounds deficient in stromule formation and chloroplast perinuclear clustering (Meier et al., 2025) will be required.

Regardless of the mechanism(s) of H_2_O_2_ movement, we observed a concurrent increase in nuclear ROS in response to PAP (Figure 1B), which could act as a potential means to elicit transcription changes for stomatal function (Pornsiriwong et al., 2017; Simeoni et al., 2022). While well beyond the scope of this study, one can speculate that chloroplast‐ or apoplast‐sourced ROS oxidizing guard cell nuclear proteins may alter protein interactions, trafficking, conformation, and function (Couturier et al., 2013; Wei et al., 2020). Mining of recent proteomics studies identifying proteins with H_2_O_2_‐sensitive cysteine residues (Huang et al., 2019; Wei et al., 2020) revealed several nuclear proteins involved in stomatal regulation (Chen et al., 2021; Tõldsepp et al., 2018). This list includes CO_2_ INSENSITIVE 1 (CIS1), which is required for elevated intracellular bicarbonate‐induced activation of S‐type anion channel currents for CO_2_‐induced stomatal closure (He et al., 2018). The diversity of these proteins opens new research avenues for understanding chloroplastic ROS signaling.

### How do ROS from different subcellular compartments intersect for stomatal closure?

Apart from direct ROS/retrograde signaling targets, an outstanding question remains: how does chloroplastic ROS intersect with apoplastic ROS? We found that PAP cannot induce ROS accumulation in the chloroplasts of *rbohD* (Figure 3B), indicating that apoplastic ROS is required for chloroplastic ROS accumulation, at least for stomatal closure. This is consistent with other findings, where chloroplasts are a site of ROS accumulation in guard cells in response to ozone, which mimics apoplastic ROS (Joo et al., 2005). Furthermore, given that PAP‐treated *rbohD* nuclei lack any ROS accumulation (Figure 3B), it is likely that apoplastic ROS also contributes, at least in part, to the PAP‐induced nuclear ROS accumulation. This could explain the nuclear ROS accumulation in PAP + DCMU‐treated wild‐type guard cells (Figure 1B).

It is unclear how PAP, ABA, or apoplastic ROS leads to perturbation of the photosynthetic electron transport chain to generate ROS in chloroplasts, but one possible pathway is via Ca^2+^ signaling or homeostasis, which can influence the activity of several PSI proteins and photoprotection mechanisms such as cyclic electron flow, thereby affecting photosynthetic ROS accumulation (Hochmal et al., 2015; Wang et al., 2019). We previously observed Ca^2+^ influx in guard cells treated with PAP (Zhao et al., 2019), although the mechanism(s) by which this occurs are unknown. The HPCA1 kinase has been identified as an apoplastic ROS sensor that activates plasma membrane calcium channels for the influx of Ca^2+^ into guard cells for stomatal closure (Wu et al., 2020). However, HPCA1 likely does not function in isolation as it acts in concert with various signaling kinases such as CBL4, CIPK26, and OST1 during systemic cell‐to‐cell ROS and Ca^2+^ signaling (Fichman et al., 2022).

Additionally, it has been proposed that chloroplast and mitochondrial ROS may be interconnected through the “malate valve,” where production of excess chloroplast ROS through the photosynthetic electron transport chain is partially ameliorated via export of reducing equivalents such as malate to the mitochondria. Malate oxidation in mitochondria releases NADH as a byproduct, with subsequent NADH oxidation leading to ROS production in mitochondria (Zhao et al., 2018, 2020). Treating guard cells with DCMU to block the accumulation of chloroplast ROS resulted in no increase in H_2_DCFDA fluorescence when measured across the entire cell (Figure 1B). By contrast, chloroplast H_2_O_2_, measured using chloroplast‐targeted roGFP‐Orp1, continued to increase even when mitochondrial ROS accumulation was chemically inhibited (Postiglione & Muday, 2023). These observations suggest that chloroplast ROS precedes mitochondrial ROS in stomatal closure, in agreement with the malate valve model, and could also explain why apoplastic ROS is required for both chloroplast ROS (Figure 3) and mitochondrial ROS (Postiglione & Muday, 2023).

We observed that chloroplast ROS is in turn required for PAP‐induced apoplastic ROS (Figure S8), which raises the possibility of a ROS positive feedback loop between the organelles and suggests retrograde signaling may occur via the apoplast as well as more directly to the nucleus. One potential mechanism is linked to the possibility of chloroplast ROS export into the cytosol and nuclei directly via aquaporins (Borisova et al., 2012) or indirectly via mitochondria as described above. Regardless of the mechanism(s) by which chloroplast ROS is relayed to the cytosol, there are multiple cytosolic and/or nuclear‐localized redox‐sensitive kinases such as OXI1 and MAP kinases (MPKs), which can activate RBOH proteins (Jalmi & Sinha, 2015).

### CPKs transcriptionally induced by PAP integrate apoplastic ROS and ion channel regulation

Apoplastic and chloroplastic ROS activate distinct signaling pathways, but high‐light stress responses initiated via the chloroplast can converge to regulate changes in transcript levels for genes related to apoplastic ROS signaling (Xu et al., 2022). Considering PAP itself is a high light stress responsive signal, it is not surprising that it upregulates CPKs capable of impacting apoplastic ROS. The timeframe of PAP‐responsive ROS burst in guard cells (10–30 min post application) is compatible with *de novo* transcription and translation of the CPKs. Nuclear mRNA transcription and accumulation of new transcripts occur within 20s of chloroplast perturbation such as light stress application (Suzuki et al., 2015; Vogel et al., 2014). Similarly, protein translation in eukaryotic cells requires just 3 min for the synthesis of a typical 50 kDa protein (Milo & Phillips, 2015).

Interestingly, some of these PAP‐induced CPKs have multiple functions in guard cells, that is, activating both RBOHD and SLAC1 (Table S1). Notably, the PAP‐induced CPKs activate SLAC1 through at least two phosphorylation sites, S59 and S120 (Figure 5B,C; Figure S12) unlike the previously reported specificity of other CPKs for S59 and OST1 for S120 (Brandt et al., 2015). The variety in target proteins and phosphosites of PAP‐induced CPKs may translate to an additive effect for stomatal regulation, since the CPKs identified to have multiple targets (i.e., CPK13, CPK32, and CPK34) provided the greatest degree of complementation in preventing leaf water loss when expressed in the *ost1* mutant background (Figure 6). The upregulation of these different CPKs that are distinct from those involved in ABA signaling identifies PAP as a node that connects the retrograde, ROS, and calcium signaling with ion channel activity for stomatal regulation.

**Figure 7:**
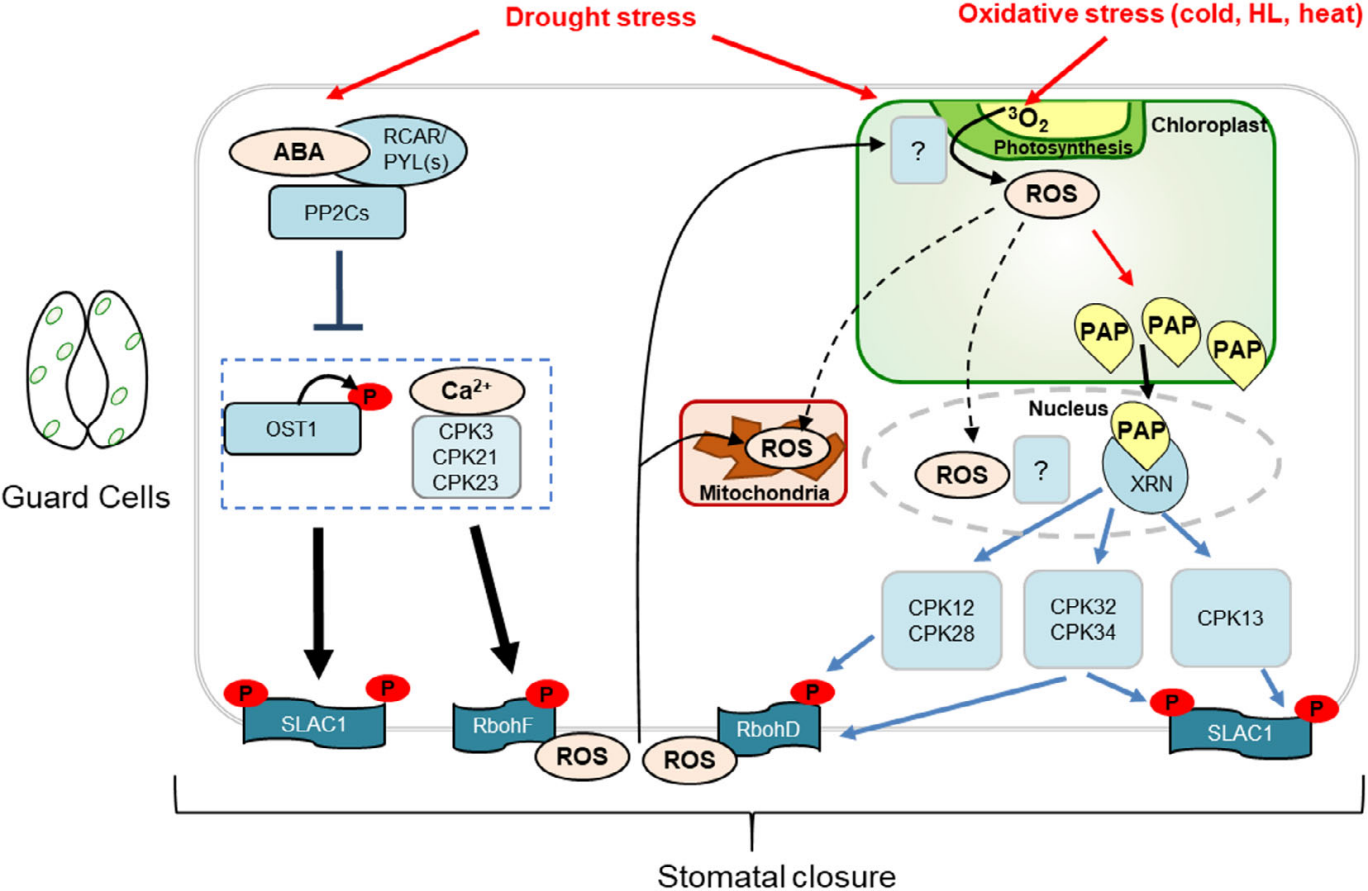
Putative model for differential interactions between PAP and ROS. In guard cells, PAP functions in parallel to ABA signaling to induce ROS production in chloroplasts, the nucleus, and the apoplast. Some of the CPKs transcriptionally induced by PAP can activate RBOHD for apoplastic ROS production, in contrast to ABA signaling via OST1 and other CPKs, which target RBOHF. Some of the PAP‐induced CPKs can activate both RBOHD and SLAC1, thus explaining the ability of PAP to function in parallel to ABA. Both chloroplast and apoplastic ROS are required for stomatal closure, and ROS production in both compartments is interdependent via unknown mechanisms. HL, highlight.

Our finding that the induction of stomatal closure by PAP occurs via RBOHD but not RBOHF, while ABA acts primarily via RBOHF (Figure 2) (Kwak et al., 2003) provides a distinct delineation between these two drought‐induced signaling molecules and their downstream pathways. During drought, ABA accumulates rapidly (~1–3 days) in *Arabidopsis* leaves compared to the slower drought‐mediated buildup of PAP (~4–9 days) (Estavillo et al., 2011). Intriguingly, plants under prolonged water stress have reduced stomatal sensitivity to ABA, which is exacerbated the longer the water stress is sustained (i.e., 24 h vs. 14 days) (Peng & Weyers, 1994): this aforementioned temporal separation of ABA and PAP accumulation may be relevant. It could lead to ABA activating particular CPKs and OST1 to produce ROS, Ca^2+^, and activate SLAC1 to close stomata in the earlier stages of drought. With prolonged drought stress, subsequent PAP accumulation activates the parallel CPK‐RBOHD‐SLAC1 pathways described herein to maintain stomatal closure. Taken together, this suggests that chloroplasts, via signals such as PAP, co‐regulate the same proteins targeted by ABA to provide a time‐gated reinforcement mechanism for stomatal regulation (Figure 7). Consequently, our results suggest the potential for PAP to intersect with ROS signaling and homeostasis in diverse cellular processes and to act in a way that builds redundancy but maintains functional distinction from ABA‐mediated ROS signaling (Figure 7).

### Is chloroplast signaling conserved across cell types?

Unanswered questions in the field of retrograde signaling revolve around functional redundancy versus specificity of signals, as well as a less commonly considered quandary, which is the spatio‐temporal intersection and neofunctionalization of the different signals in specialized cells. There is an intriguing “sensory plastid” hypothesis of cell type‐specific functionalization of chloroplasts, where the specialized chloroplast or plastid will have a specialized environmental sensing role, depending on which cell type it is localized in (Mackenzie & Mullineaux, 2022). SAL1 is expressed in multiple leaf cell types including vascular bundles, mesophyll, and guard cells (Estavillo et al., 2011; Wang et al., 2023), but PAP appears to function differently across the leaf, at least with respect to ROS. PAP is a chloroplast ROS‐triggered signal in foliar tissue (Chan, Mabbitt, et al., 2016), and its accumulation results in the up‐regulation of H_2_O_2_ scavengers, such as *APX2* in this tissue type, that collectively result in the quenching of excess ROS in vascular bundles (Figure S4) (Estavillo et al., 2011). In direct contrast, PAP induces the induction of chloroplast PSI ROS in guard cells (Figure 1; Figure S4). Whether these differential effects of PAP are simply correlated or truly linked to chloroplast and cell‐type specialization will require investigation. Notably, a technical limitation that will need consideration in future studies is that fluorescein‐based dyes like H_2_DCFDA are susceptible to multiple factors, including local O_2_ levels, photooxidation, and alkaline hydrolysis, necessitating rigorous controls (Murphy et al., 2022; Yu et al., 2021). In our experiments, we accounted for photooxidation by imaging H_2_DCFDA fluorescence at identical time intervals across mock (Control)‐, ABA‐, or PAP‐treated guard cells (Figures 1 and 3); and utilized a separate probe, PO1 (Figure S3), which is boronate‐based (Dickinson et al., 2010). The pH sensitivity of H_2_DCFDA also requires consideration since ABA‐responsive stomatal closure involves rapid cytosolic alkalinization preceding ROS production, whereas flg22‐responsive stomatal closure does not (Li et al., 2021). Whilst PAP‐induced stomatal closure is ABA‐independent (Pornsiriwong et al., 2017) and involves flg22‐responsive components such as RBOHD (Figure 3), future research should determine whether pH also plays a role in PAP signaling across the different subcellular compartments. Parallel analysis of multiple transgenic lines expressing pH‐insensitive ROS biosensors roGFP2‐Orp1 or Hyper7 (Ugalde et al., 2021), as well as pH biosensors such as CapHensor (Li et al., 2021) targeted to different subcellular compartments and in different cell types, will assist in determining the true extent and dynamics of cell‐specialized PAP‐ROS interactions for stress acclimation.

In conclusion, while in vascular bundles the upregulation of ROS quenching processes is mediated by the retrograde signal 3′‐phosphoadenosine 5′‐phosphate (PAP), here we show that PAP induces ROS accumulation for stomatal closure, demonstrating how chloroplast signals can intersect in a cell‐specific manner with subcellular ROS networks for cellular stress responses.

## MATERIALS AND METHODS

### Plant material

The *Arabidopsis* wild type used in this study is Columbia‐0 (Col‐0). Mutants and transgenic lines are in the Col‐0 background. Lines used in this study are *sal1*‐6 (SALK_020882 [Estavillo et al., 2011]); *sal1*‐8 (*alx8*) (Wilson et al., 2009), tAPX OE (Murgia et al., 2004); *rbohD* (CS10555 [Torres et al., 2002]); *rbohF* (CS10557 [Torres et al., 2002]); *rbohDF* (CS10558 [Torres et al., 2002]); *slac1*‐4 (SALK_137265 [Vahisalu et al., 2008]); *slac1‐*1, *slac1*‐S59A, *slac1*‐S120A, and *slac1* S59A/S120A (Brandt et al., 2015); and *ost1*‐3 (SALK_008068 [Yoshida et al., 2002]). Candidate CPKs were transformed in the Col‐0 *ost1* mutant using the agrobacterium transformation floral dip method (Clough & Bent, 1998) and hygromycin screen (Harrison et al., 2006); candidates were cloned in the pMDC32 binary vector (Curtis & Grossniklaus, 2003) with the cauliflower mosaic virus 35S promoter replaced with the guard cell promoter pGC1 (Yang et al., 2008). For stomatal aperture and water‐loss assays, *Arabidopsis* plants were grown on soil with 16 h of light at 22°C, except for the *rboh* mutants and the respective Col‐0 control, which were grown with 12 h of light. For confocal microscopy and ozone treatment assays, *Arabidopsis* plants were grown on soil in 12 h of light.

### Ozone treatment

Three‐week‐old *Arabidopsis* plants were exposed to 50 ppb ozone for 6 h from 9:00 to 15:00. Plants were then transferred back into the growth chamber, with damage visually being scored and photographed 24 h post‐exposure. For electrolyte leakage measurements, whole rosettes were collected after the end of the ozone exposure (both‐ozone treated and those in the control condition, i.e., plants left in the growth chamber at <20 ppb ozone) and placed into 20 mL of MilliQ H_2_O. Samples were measured using a Mettler conductivity meter (Model FE30) to obtain ion readings 24 h post the initial ozone exposure. Samples were frozen completely, defrosted, and then measurements were taken again to obtain total ion content, with values plotted as a % total ion lost.

### Stomatal aperture assay

Stomatal aperture assays were performed as per Pornsiriwong et al. (2017). For any epidermal peel assay, samples were harvested from 3‐ to 5‐week‐old plants, with a minimum of three biological replicates used per treatment and genotype. The epidermal layer was gently adhered adaxial side down to the bottom of the experimental chamber (i.e., small petri dish) coated with optically clear and pressure‐sensitive silicon adhesive (Factor II Inc.). Samples were incubated in Opening Buffer [50 mM KCl, 5 mM MES titrated to pH 6.1 with NaOH] for 2 h and then transferred to an inverted bright‐field microscope capable of 400× magnification. An initial image was taken and then another after 10 min to ensure the stomata were open before subsequent assays. Samples were then washed gently three times with Measuring Buffer [10 mM KCl, 5 mM MES titrated to pH 6.1 with Ca(OH)_2_] and then treated with the appropriate chemical dissolved in Measuring Buffer, with images taken every 10 min. Stomatal aperture data are expressed as relative change over the control period (i.e., change from the initial image taken before washes and treatment) after 20 min. The stomatal pore area was determined using Fiji (NIH) where pore areas were converted to pixels, and the relative change in pixels per pore area between images was compared.

### Microscopy for epidermal peels

Quantification of ROS in guard cells of epidermal peels was measured using 20 μM 2′,7′‐dichlorodifluorescein diacetate (H_2_DCFDA; Invitrogen) according to Pornsiriwong et al. (2017), with epidermal peels treated as per Pornsiriwong et al. (2017). Fluorescence images were taken using a Leica inverted confocal microscope using a 496‐nm excitation line of an argon multiline laser. H_2_DCFDA fluorescence emission was detected at 505–525 nm, while chloroplast autofluorescence was detected at 680–700 nm. Images were quantified using Fiji (NIH), with the relative change in fluorescence of ROS determined with images before and after treatment. ROIs were drawn for specific organelles and kept consistent between images to determine changes in organelle ROS levels.

Visualization of intracellular and apoplastic ROS in guard cells was performed using 20 μM H_2_DCFDA or 100 μM peroxy orange 1 for H_2_O_2_ (PO1; Merck Sigma‐Aldrich) and 100 μg mL^−1^ Oxyburst Green H2HFF‐BSA (Thermo Scientific), respectively. Markers for chloroplasts and nuclei were chlorophyll fluorescence, 5 μM Hoescht (Hoescht 33 258; Merck Sigma‐Aldrich), and 500 μM 4′,6‐Diamidino‐2‐phenylindole dihydrochloride, 2‐(4‐Amidinophenyl)‐6‐indolecarbamidine dihydrochloride (DAPI dihydrochloride; Merck Sigma‐Aldrich), respectively. Qualitative visualization experiments were performed using a Leica DM5500 epifluorescence microscope, with appropriate filter cubes for the respective fluorescent markers.

### HEK293T RBOHD ROS assay

A prepared 96‐well plate with HEK293T cells transfected with genes of interest (see Extended methods for cloning specifics, transfection protocol, and protein extraction and western blot associated, adapted from Kimura et al., 2022) was gently washed with 100 μL Hank's Balanced Salt Solution (HBSS, with Ca^2+^ and Mg^+^; ThermoFisher Scientific), and then 100 μL of the assay buffer (29.9 mL HBSS, with Ca^2+^ and Mg^+^, 90 μL of 0.002 g horseradish peroxidase dissolved in 100 μL HBSS solution, 18.8 μL of 0.4 M luminol) was added to each well. The plate was put into a plate reader capable of polarization mode dispersion to measure luminescence at 37°C. First, luminescence was measured for 15 min for cells only in the assay buffer (measurement time 1 sec/well, interval 1 min/well), 50 μL of 3 μM ionomycin solution dissolved in the assay buffer was dispensed in each well and measured for another 15 min.

### Electrophysiology

SLAC1‐mediated anion currents in oocytes were performed as per Pornsiriwong et al. (2017). See Extended Methods.

### Statistical analyses

Data presented as boxplots have the middle line marking the median, the box indicating the upper and lower quartiles, and the whiskers showing the minimum and maximum values within 1.5× the interquartile range. Data were analyzed using RStudio 2021.09.01 Build 351/R version 4.0.3. For ion leakage assay after ozone treatment, stomatal aperture assays, ROS changes in guard cell components or whole stomata, HEK293T ROS assay, and electrophysiology measurements showing SLAC1 anion currents at −130 Mv, a linear model (with independent factors specified in figure legends) was applied using the R package *lmerTest*. For experiments where multiple replicates were taken from a given biological replicate (i.e., different stomata from a biological replicate), the random effect was “biological replicate.” The ANOVAs specified are ANOVA Satterthwaite's Method, with significant differences between factors (denoted in figure legends) determined by post hoc Tukey HSD using the R package *emmeans*.

## AUTHOR CONTRIBUTIONS

EET, SJF, SK, MC, MW, SB, CF, JK, Z‐HC, BJP, and KXC designed research; EET, SJF, HMV, CZ, AB, and KXC performed research; EET, SJF, HMV and analyzed data; EET, BJP, and KXC wrote the paper with contributions from all authors.

## CONFLICT OF INTEREST

No competing interests to declare.

## Supporting information


**Figure S1.** PAP treatment induces a ROS burst in nuclei of guard cells as indicated by co‐localization with Hoescht.
**Figure S2.** PAP treatment induces a ROS burst in nuclei of guard cells as indicated by co‐localization with DAPI.
**Figure S3.** PAP treatment increases H_2_O_2_‐responsive peroxy orange 1 (PO1) fluorescence in guard cells.
**Figure S4.** Constitutive PAP accumulation has different effects on ROS accumulation in different tissue/cell types.
**Figure S5.** Reduction of effective PSII quantum yield (Y(II)) in mesophyll and epidermal tissue by DCMU.
**Figure S6.** PAP‐ and ABA‐mediated stomatal closure are differentially inhibited by various concentrations of DCMU.
**Figure S7.** Inhibition of chloroplast ROS production with DCMU diminishes PAP‐induced apoplastic ROS production.
**Figure S8.** Retrograde chloroplast signaling via SAL1‐PAP complements apoplastic signaling mediated by OST1.
**Figure S9.** Expression of CPKs and RBOHD in HEK293T cells.
**Figure S10.** Current traces of CPK candidates.
**Figure S11.** Representative current traces of CPK13 with different mutated phosphosite SLAC1.
**Figure S12.** slac1‐4 mutants are unresponsive to ABA and PAP.
**Table S1.** Summary of PAP‐transcriptionally induced candidate function in RBOHD and SLAC1 activity and water‐loss assay in the *ost1* background.
Extended Material and Methods.

## Data Availability

The data that support the findings of this study are available in the Supplementary Material of this article.
