# Supplementary material for: SAL1‐PAP retrograde signaling orchestrates photosynthetic and extracellular reactive oxygen species for stress responses
Source: Plant J. 2025 Jun 12;122(5):e70271. doi: 10.1111/tpj.70271 (PMC12161943; doi:10.1111/tpj.70271)
Supplement: Supplementary file 1 — Figure S1. PAP treatment induces a ROS burst in nuclei of guard cells as indicated by co‐localization with Hoescht. Figure S2. PAP treatment induces a ROS burst in nuclei of guard cells as indicated by co‐localization with DAPI. Figure S3. PAP treatment increases H2O2‐responsive peroxy orange 1 (PO1) fluorescence in guard cells. Figure S4. Constitutive PAP accumulation has different effects on ROS accumulation in different tissue/cell types. Figure S5. Reduction of effective PSII quantum yield (Y(II)) in mesophyll and epidermal tissue by DCMU. Figure S6. PAP‐ and ABA‐mediated stomatal closure are differentially inhibited by various concentrations of DCMU. Figure S7. Inhibition of chloroplast ROS production with DCMU diminishes PAP‐induced apoplastic ROS production. Figure S8. Retrograde chloroplast signaling via SAL1‐PAP complements apoplastic signaling mediated by OST1. Figure S9. Expression of CPKs and RBOHD in HEK293T cells. Figure S10. Current traces of CPK candidates. Figure S11. Representative current traces of CPK13 with different mutated phosphosite SLAC1. Figure S12. slac1‐4 mutants are unresponsive to ABA and PAP. Table S1. Summary of PAP‐transcriptionally induced candidate function in RBOHD and SLAC1 activity and water‐loss assay in the ost1 background. Extended Material and Methods. [file TPJ-122-0-s001.pdf]

SAL1-PAP retrograde signaling orchestrates photosynthetic and extracellular reactive oxygen species for stress responses

Estee E. Tee[1,5], Stephen J. Fairweather[1], Hanh M. Vo[1,6], Chenchen Zhao[2,7], Andrew Breakspear[3], Sachie Kimura[4,8], Melanie Carmody[4,9], Michael Wrzaczek[4,10,11], Stefan Bröer[1], Christine Faulkner[3], Jaakko Kangasjärvi[4], Zhong-Hua Chen[2], Barry J. Pogson[1*], Kai Xun Chan[1*]

[1]Research School of Biology, Australian National University, 2601 Canberra, Australia
[2]School of Science, Western Sydney University, 2753 Sydney, Australia
[3]Cell and Developmental Biology, John Innes Centre, NR4 7UH Norwich, United Kingdom
[4]Organismal and Evolutionary Biology, Viiki Plant Science Centre, University of Helsinki, 00014 Helsinki, Finland
[5]Present address: Cell and Developmental Biology, John Innes Centre, NR4 7UH Norwich, United Kingdom
[6]Present address: School of Natural Sciences, University of Tasmania, 7001 Hobart, Australia
[7]Present address: Tasmanian Institute of Agriculture, University of Tasmania, 7250 Launceston, Australia
[8]Present address: NAPIGEN, Inc., 19803 Delaware, United States of America
[9]Present address: Synthetic Biology Future Science Platform, Commonwealth Scientific and Industrial Research Organisation, 2601 Canberra, Australia
[10]Present address: Institute of Plant Molecular Biology, Czech Academy of Sciences, 370 05 České Budějovice, Czech Republic
[11]Present address: University of South Bohemia, Faculty of Science, Department of Experimental Plant Biology, 370 05 České Budějovice, Czech Republic

[*] **Authors for Correspondence:** kai.chan@anu.edu.au and barry.pogson@anu.edu.au

This PDF file includes:

Extended Material and Methods

Figures S1 to S12

Table S1

SI References

Extended Material and Methods

HEK293T cloning vectors and transfection

CPK genes were cloned into pEF1-MCS-3Myc, an altered pEF1/*myc*-His vector (Invitrogen)(Kimura et al. 2020). The vector with RBOHD, pcDNA3.1-3FLAG-RBOHD, was previously described (Kaya et al. 2019). A midiprep for DNA used for transfecting HEK293T plasmids was prepared using the NucleoBond® Xtra Midi Plus Kit (MACHEREY-NAGAL GmbH & Co). HEK293T cells were sub-cultured for four weeks, with transfection and ROS assays performed on cells two to four weeks of age. A cell suspension was adjusted to a cell density of 1.0-2.0 × 10^5 cells/mL, then 130 µL was aliquoted into each well of a white Corning® BioCoat® Poly-D-lysine coated 96 well plate, then incubated at 37 °C 5% CO_2 for 12-24 hours. A plasmid ratio of 5:1 co-transfection ratio of pcDNA3.1 vector:pEF1/*myc*-His vector was used (100 ng to 20 ng). For each combination tested, 12 wells were transfected with the exception of the non-transfection control. Plasmid DNA was diluted to be 100 ng/µL so total DNA content added was 1.2 µL per well. 6.3 µL Opti-MEM (ThermoFisher Scientific) was transferred into sterile microcentrifuge tubes, and 0.36 µL per well to be transfected of GeneJuice® Transfection Reagent (Novagen®, Millipore) was added directly to the Opti-MEM, mixed thoroughly by vortexing. The mixture was incubated at room temperature for five minutes, then plasmid DNA was added, mixed by gently pipetting and incubated further for another five minutes. The mixture was added dropwise to prepared cells, and the whole plate was gently rocked to ensure even distribution. Cells were then incubated for another 48 hours at 37 °C 5% CO_2. CPK candidates were screened either in combination with the pcDNA3.1 empty vector of RBOHD for a minimum of three separate plates. Data per plate was from three specific wells, with each plate designed so there were three replicates per transfection combination, and an average measured between the three.

Protein extraction and western blot for HEK293T cells

The medium was removed from three selected wells from each transfection combination. 50 µL of protein sampling buffer [50 mM Tris-HCl, 2% SDS, 10% glycerol, 10% 2-mercaptoethanol, 300 mM DTT] was added and left to incubate for five minutes. The lysates were collected into microcentrifuge tubes, and before protein loading, samples were pipette syringed to fragment genomic DNA. For HEK293T cells transfected with plasmids containing the FLAG tag, protein amount loaded was 10 µL, while plasmids containing the *myc* tag, protein amount loaded was 50 µL with the exception of GFP, in which only 5 µL was loaded. A wetblot transfer onto Immobilon® Transfer Membranes (Merck Millipore) was performed, and membranes were probed with anti-FLAG (1:3000 dilution) and anti-β ACTIN (1:5000 dilution), or anti-c-Myc (1:3000 dilution) followed by IRDye 800 anti-mouse-IgG (IRDye ® 800CW Goat anti-Mouse IgG (H + L), 0.5 mg; LI-COR Biosciences).

Xenopus Laevis *surgery and oocyte preparation*

Female *Xenopus laevis* frogs were anaesthetized by submersion for 30 min in 3-Aminobenzoic acid ethyl ester (1.5 g/L) (Sigma-Aldrich), then placed on ice to slow blood flow; surgery was conducted as previously described (Fairweather et al. 2015; Fairweather et al. 2021). Ovary sac sections were maintained in OR^{2-} buffer (82.5 mM NaCl, 2.5 mM KCl, 1 mM $MgCl_2$, 1 mM Na_2HPO_4, 5 mM HEPES-NaOH, pH 7.8) in sterile Petri dishes and cut into small clumps of ~30-50 oocytes. Digestion of folliculated oocytes into individually accessible de-folliculated oocytes was conducted by incubating cut ovary sections in 1.5 mg/mL collagenase D (Sigma-Aldrich) dissolved in OR^{2-} (pH 7.8) buffer for 2 hours at 28 °C followed by 4 hours at 18 °C. The success of digestion and de-folliculation was assessed following the washing and examination of oocytes using 2 L of OR^{2-} buffer followed by 2 L OR^{2+} buffer (OR^{2-} supplemented with 1.5 mM $CaCl_2$ and 50 µg/mL gentamycin, pH 7.8). Selected healthy oocytes were maintained at 16−18 °C in OR^{2+} until required for electrophysiology experiments. Maintenance of animals and preparation of oocytes was approved by the Australian National University animal ethics review board (ANU Protocol A2017/36).

Electrophysiology recordings

New CPKs of interest were first cloned in pCR^TM8/GW/TOPO vector and subcloned into a destination pGEM-HE oocyte vector used in Pornsiriwong et al. (2017). For SLAC1 phosphosite mutations, site-directed mutagenesis was performed using the QuikChange XL Site-Directed Mutagenesis Kit (Agilent Technologies) [for SLAC1-S120A: GCAAACAAAAG**G**CTTTATTGCCTTCTAT and ATAGAAGGCAATAAAG**C**CTTTTGTTTGC; for SLAC1-S59A: GCAGACAGGTTGCGCTAGAGACAGG and CCTGTCTCTAGCG**C**AACCTGTCTGC]. Plasmids were first linearization with either restriction enzymes NheI or SbfI, followed by cRNA synthesis by mMESSAGE mMACHINE® T7 kit (Thermo Fisher Scientific). Prepared *Xenopus laevis* oocytes were injected with RNase-free H_2O, or cRNA. For kinase or SLAC1/SLAC1 mutant controls, 25 ng was injected. SLAC1/SLAC1 mutants were mixed with individual CPKs for a ratio of 2:1. Whole-cell currents were recorded from oocytes 2-3 days post injection using an Axoclamp 2B two-electrode clamp circuit. Measurements were recorded in an I_{anion} measuring solution [48 mM NaCl, 48 mM CsCl, 1 mM $MgCl_2$, 1 $CaCl_2$, 10 mM MES, titrated to pH 5.6 with 5 M HCl], with the following protocol: holding at 0 mV for 1 s, testing from +50 to -130 mV for 10 × 10 s cycles (holding voltage taken every 20 mV intervals), and holding at 0 mV for 1 s. Two-electrode voltage-clamp recordings were performed with 1 × LU and 10 × MGU head stages connected to a Geneclamp 500B electronic amplifier (Axon Instruments, Union City, U.S.A). The output signal was amplified 10 times and filtered at 1 kHz, with the analogue signal converted into digital by a Digidata 1322A (Axon Instruments). Data were sampled at 10 Hz using pCLAMP software (Axon Instruments), and steady state readings were taken as an average at the end of the 10 second holding voltage step using the program Clampfit 10.7.0.3 (Molecular Devices).

3,3'-diaminobenzidine (DAB) staining for ROS

One fully expanded leaf per plant was excised at the petiole from three week-old wild type and *sal1-8* plants. The petiole was immersed into 700μL of 5mM 3,3'-diaminobenzidine (DAB) solution, pH 3.8 layered onto 900μL of solidified 1% agarose in a 1.5 mL Eppendorf tube. The leaves were held in place using parafilm at the opening of the Eppendorf tube, then incubated in the dark for 15 minutes to allow uptake of the DAB solution. The leaves were then exposed to either 1 hour of growth light (120 μmol m-2 s-1) or high light (1000 μmol m-2 s-1). Chlorophyll was then removed by immersion in 100% ethanol overnight. Leaves were then rehydrated in 1 mL of 40% glycerol prior to imaging in 24-well plates.

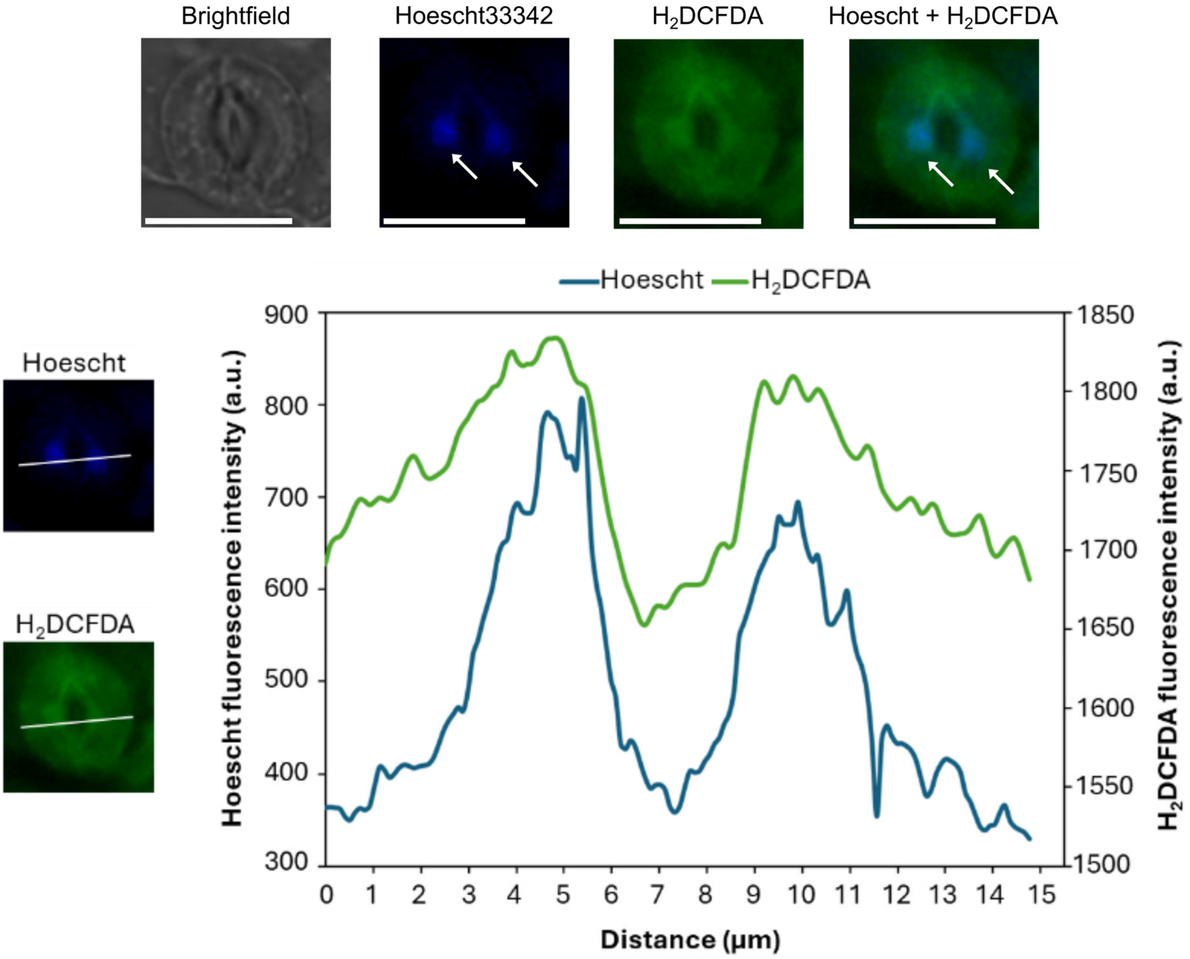

Supplementary Figure S1. PAP treatment induces a ROS burst in nuclei of guard cells as indicated by co-localization with Hoescht. (A) Co-localisation of PAP-induced ROS was visualised in guard cells co-treated with 100 μM PAP, 5 μM Hoescht and 20 μM H_2DCFDA. Guard cells were treated with PAP for 30 min prior to visualisation of Hoescht and H_2DCFDA fluorescence. Nuclei are indicated by white arrows. Scale bar = 12 μm.

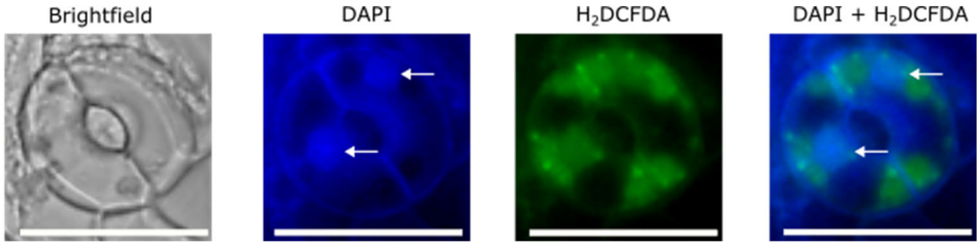

Supplementary Figure S2. PAP treatment induces a ROS burst in nuclei of guard cells as indicated by co-localization with DAPI. Co-localization of PAP-induced ROS was visualised in guard cells co-treated with 100 μM PAP, 500 μM DAPI and 20 μM H_2DCFDA. Guard cells were treated with PAP for 30 min prior to visualisation of DAPI and H_2DCFDA fluorescence. Nuclei are indicated by white arrows. Scale bar = 12 μm.

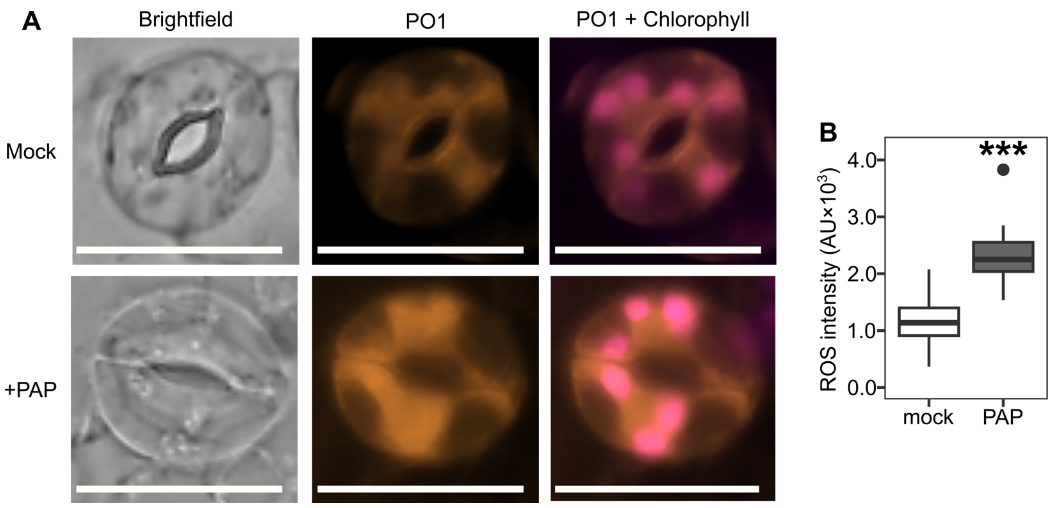

Supplementary Figure S3. PAP treatment increases H_2O_2-responsive peroxy orange 1 (PO1) fluorescence in guard cells. Representative images for guard cells (A) cotreated with 100 µM PO1 and either mock or 100 µM PAP for 30 min in the light, with corresponding quantification (B). Each condition contains a minimum of three wild type plants with a minimum of n ≥ 10 stomata per treatment combination. Treatment significantly impacted fluorescence (ANOVA; F=67.4, df=1, p<0.001). Significant difference to mock treatment denoted by ***. Scale bar = 12 µm.

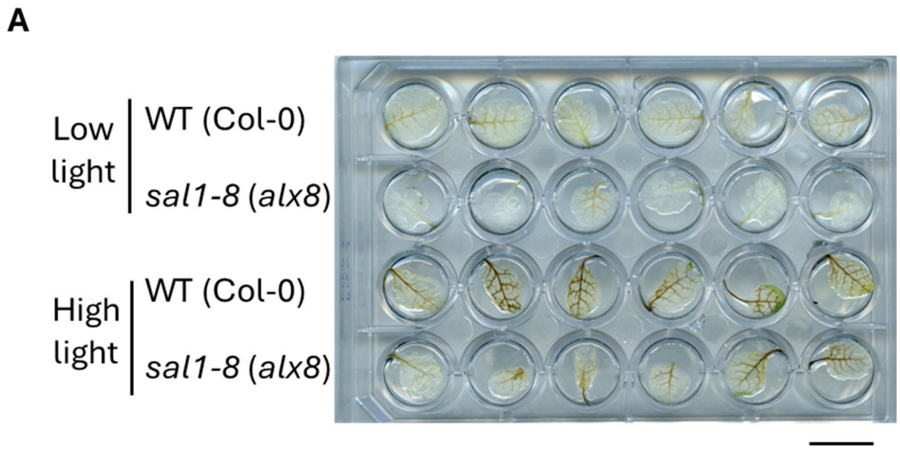

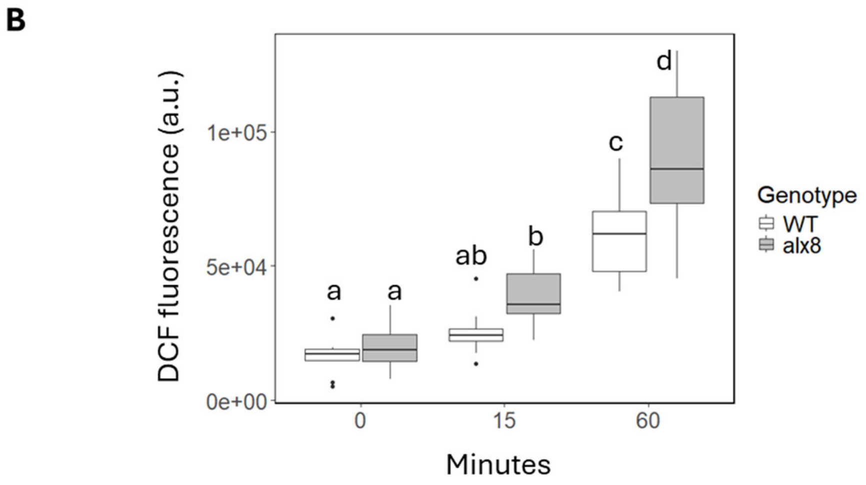

Supplementary Figure S4. Constitutive PAP accumulation has different effects on ROS accumulation in different tissue/cell types. (A) ROS-responsive 3, 3'-diaminobenzidine staining of wild type (WT) and *sal1*-8 leaves exposed to 1 h of either low light (120 µmol m^{-2} s^{-1}) or high light (1000 µmol m^{-2} s^{-1}). Each well contains a fully expanded leaf from a distinct individual plant, i.e. n=6 biological replicates per genotype per treatment. Scale bar = 1.55 cm. (B) ROS-responsive H_2DCFDA fluorescence in wild type and *sal1*-8 guard cells after 0, 15 and 60 min of 100 µM ABA treatment. n =9-25 stomata per timepoint. a.u.= arbitrary units. Statistically significant differences are indicated by a,b,c ($p<0.05$, ANOVA Tukey HSD).

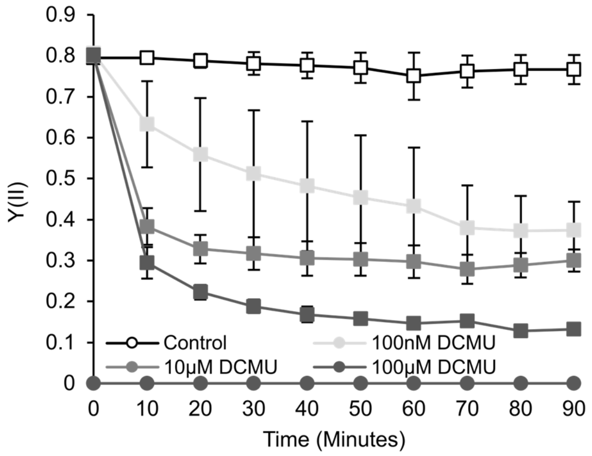

Supplementary Figure S5. Reduction of effective PSII quantum yield (Y(II)) in mesophyll and epidermal tissue by DCMU. Reduction of Y(II) was monitored over time in response to three concentrations (100 nM, 10 µM and 100 µM) of DCMU. Mesophyll tissue is represented in squares while epidermal tissue is represented in circles. Values are averages of three replicates per treatment with error bars representing SD.

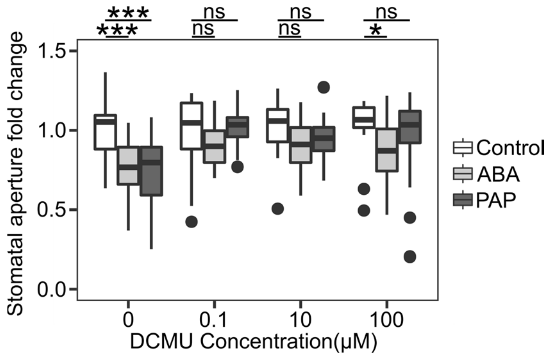

Supplementary Figure S6. PAP and ABA-mediated stomatal closure is differentially inhibited by various concentrations of DCMU. Stomatal closure in response to 10 min treatment of 100 μM ABA or 100 μM PAP with 0, 0.1, 10 or 100 μM DCMU. Each condition contains a minimum of three wild type plants with a minimum of n ≥ 26 stomata per treatment combination. 'DCMU' and 'Treatment' significantly impacted closure with a significant interaction between the two (ANOVA; $F=13.3$, $df=3$, $p<0.001$; $F=22.4$, $df=2$, $p<0.001$; $F=4.1$, $df=6$, $p<0.001$). Significant differences from respective DCMU Control denoted by *, $p<0.05$ or ***, $p<0.001$.

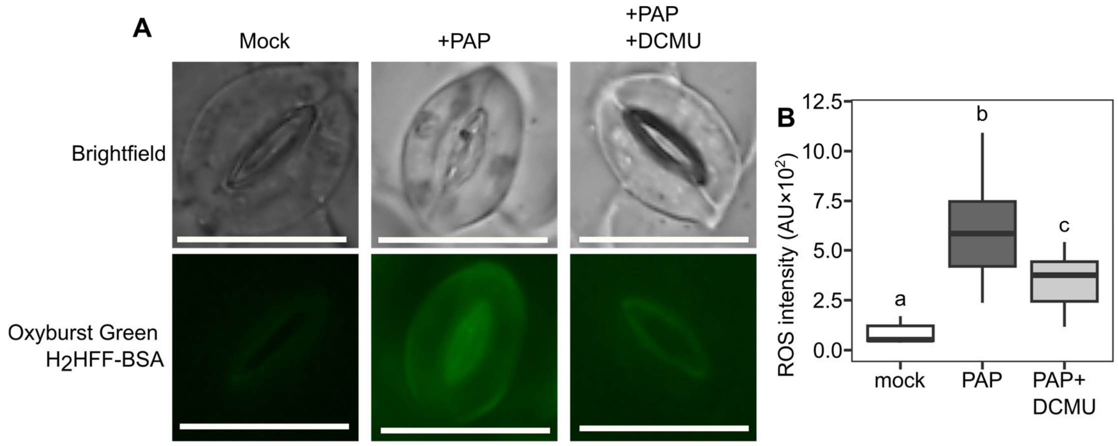

Supplementary Figure S7. Inhibition of chloroplast ROS production with DCMU diminishes PAP-induced apoplastic ROS production. Representative images for guard cells (A) treated with either mock, 100 μM PAP, or 100 μM PAP and 10 μM DCMU, with corresponding quantification (B). All treatments were co-incubated in the light for 20 min with 100 μg/ml Oxyburst Green H2HFF-BSA. Treatment significantly impacted fluorescence (ANOVA; F=26.7, df=2, p<0.001). Significant differences denoted by a, b and c. Each condition contains a minimum of three wild type plants with a minimum of n ≥ 20 stomata per treatment combination. Scale bar = 18 μm.

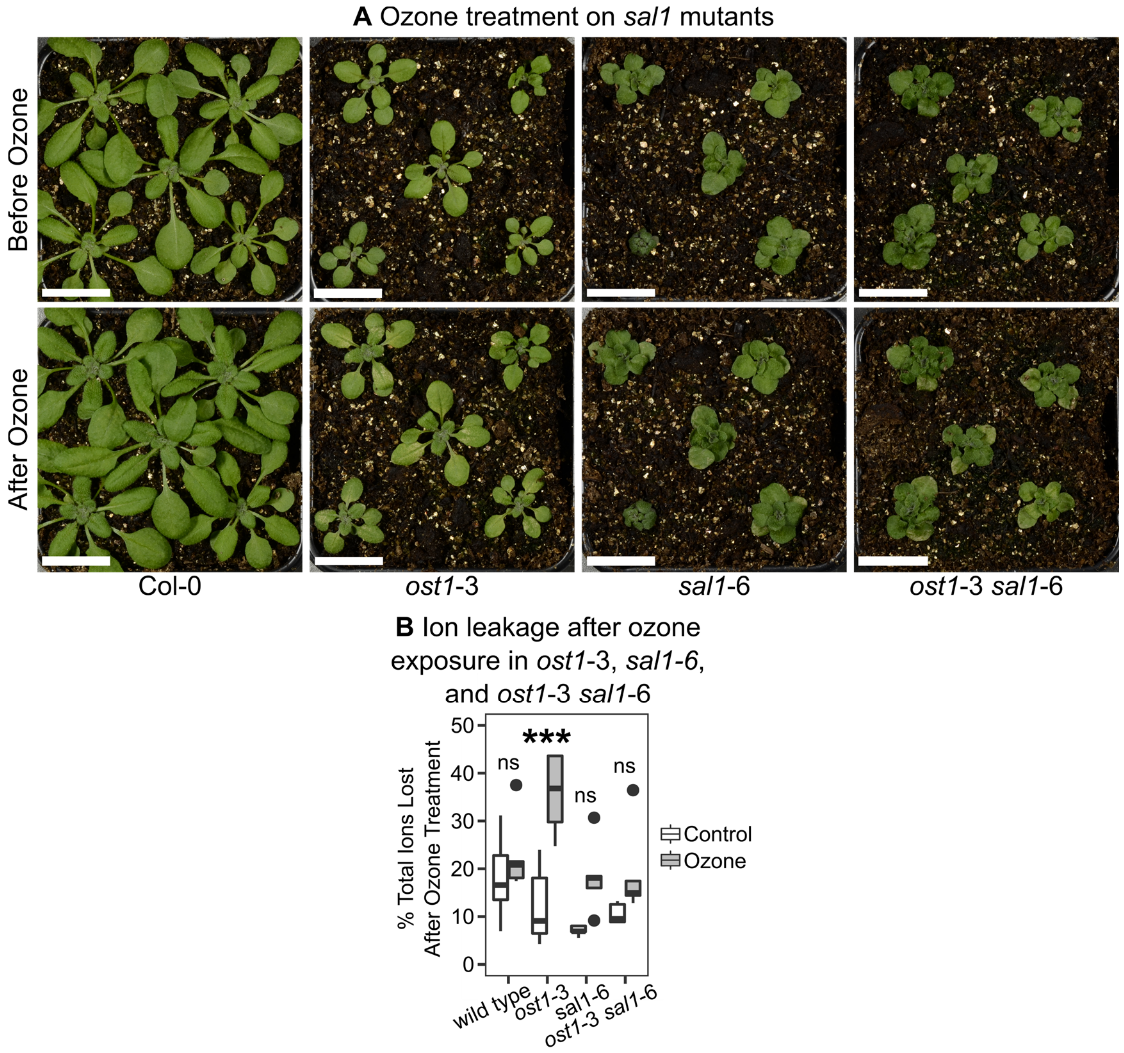

Supplementary Figure S8. Retrograde chloroplast signaling via SAL1-PAP complements apoplastic signaling mediated by OST1. (A) Pictures of 3-week-old plants, taken before and after 24 hours post the end of a six-hour exposure to 350 ppb ozone (i.e., 30 hours post initial exposure). Scale bar = 2 cm. (B) Electrolyte leakage measurements from plants harvested after six-hour exposure to ozone or control conditions, 24 hours after start of treatment. Values are plotted as a % total ion lost, with n = 5 per genotype and treatment. Factors 'Genotype' and 'Treatment' had a significant impact on total ion lost (ANOVA; F=4.7, df=3, p<0.01 and F=25.5, df=1, p<0.001), with significant differences between treatments within a genotype denoted by ***, p<0.001.

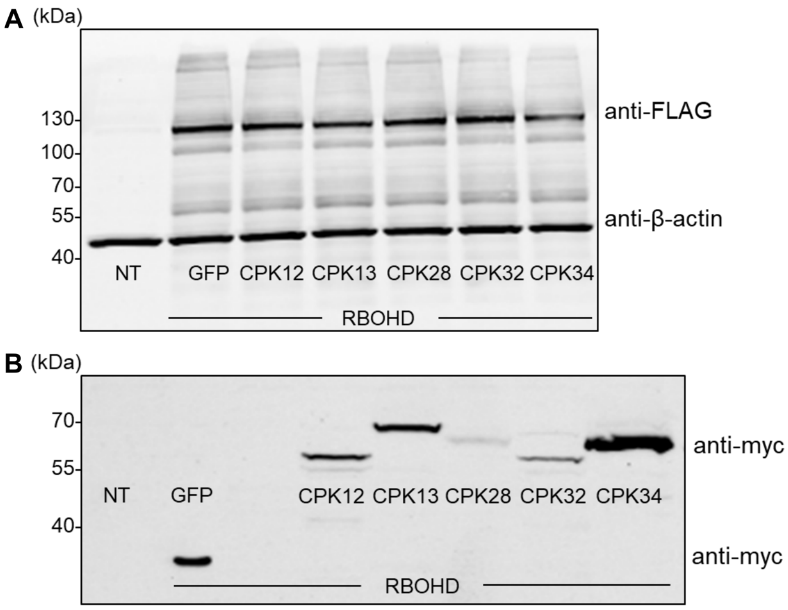

Supplementary Figure S9. Expression of CPKs and RBOHD in HEK293T cells. (A) Example western blots showing equal protein loading of total protein via anti-β-actin. NT stands for non-transfected control. Equal expression of RBOHD (anti-FLAG), associated with Fig. 5. (B) Protein expression indicated by anti-myc, fused to either GFP or the CPKs. There was unequal protein expression of the CPKs as indicated by anti-myc despite equal total protein loading as indicated by (A).

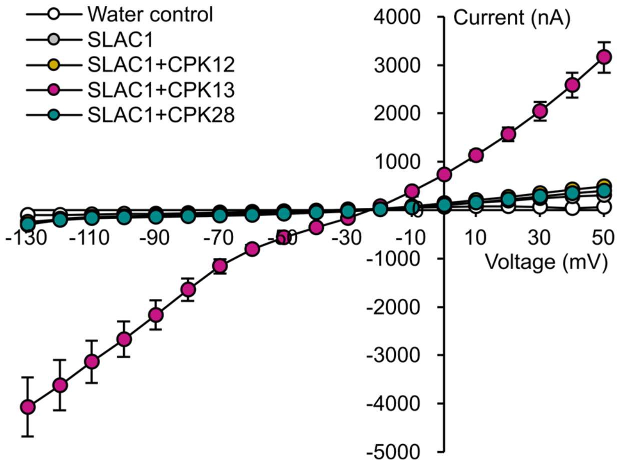

Supplementary Figure S10. I/V plots of CPK candidates. I/V plots of CPK12, CPK13 and CPK28 activations of SLAC1 anion channel in oocytes. Oocytes were co-injected with the kinase and the channel SLAC1. Values are means of four to eight oocytes ± SEM. Anion channel activity shown as current data at voltage pulses ranging from +50 to -130 mV in 10mV decrements.

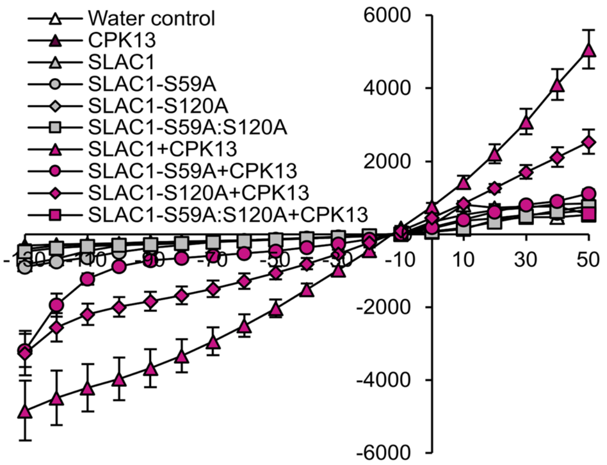

Supplementary Figure S11. Representative I/V plots of CPK13 with different mutated phosphosite SLAC1. I/V plots of CPK13 with mutated SLAC1 anion channel in oocytes. Oocytes were injected with CPK13 alone, the channel SLAC1 (wild type or mutated), H_2O (Water control), or in combination. Values are means of a minimum of six oocytes per combination ± SEM. Anion channel activity shown as current data at voltage pulses ranging from +50 to -130 mV in 10 mV decrements.

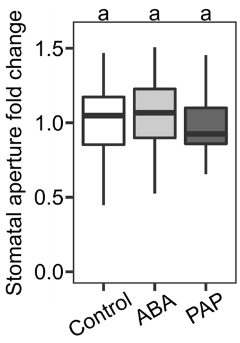

Supplementary Figure S12. *slac1*-4 mutants are unresponsive to ABA and PAP. Stomatal closure of *slac1*-4 mutants in response to 10 min treatment of 100 μM ABA or 100 μM PAP. Each treatment contains five biological replicates with a minimum of n > 36 stomata per treatment. 'Treatment' did not impact closure (ANOVA; F=1.3, df=2, p=0.3).

Table S1. Summary of PAP-transcriptionally induced candidate function in RBOHD and SLAC1 activity, and water-loss assay in *ost1* background.

Gene	RBOHD	SLAC1	Complementation of *ost1*
CPK12	Yes	No	Minor
CPK13	No	Yes	Partial
CPK28	Yes	No	n/a
CPK32	Yes	Yes	Partial
CPK34	Yes	Yes	Partial

SI References

Fairweather SJ, Bröer A, Subramanian N, Tumer E, Cheng Q, Schmoll D, O'Mara ML, Bröer S. 2015. Molecular basis for the interaction of the mammalian amino acid transporters B0AT1 and B0AT3 with their ancillary protein collectrin. Journal of Biological Chemistry. 290(40):24308–24325. doi:10.1074/jbc.M115.648519.

Fairweather SJ, Okada S, Gauthier-Coles G, Javed K, Bröer A, Bröer S. 2021. A GC-MS/Single-Cell Method to Evaluate Membrane Transporter Substrate Specificity and Signaling. Front Mol Biosci. 8:140. doi:10.3389/FMOLB.2021.646574/BIBTEX.

Kaya H, Takeda S, Kobayashi MJ, Kimura S, Iizuka A, Imai A, Hishinuma H, Kawarazaki T, Mori K, Yamamoto Y, et al. 2019. Comparative analysis of the reactive oxygen species-producing enzymatic activity of Arabidopsis NADPH oxidases. Plant J. 98(2):291–300. doi:10.1111/TPJ.14212.

Kimura S, Hunter K, Vaahtera L, Tran HC, Citterico M, Vaattovaara A, Rokka A, Stolze SC, Harzen A, Meißner L, et al. 2020. CRK2 and C-terminal Phosphorylation of NADPH Oxidase RBOHD Regulate Reactive Oxygen Species Production in Arabidopsis. Plant Cell. 32(4):1063–1080. doi:10.1105/TPC.19.00525.

Pornsiriwong W, Estavillo GM, Chan KX, Tee EE, Ganguly D, Crisp PA, Phua SY, Zhao C, Qiu J, Park J, et al. 2017. A chloroplast retrograde signal, 3'phosphoadenosine 5'-phosphate, acts as a secondary messenger in abscisic acid signaling in stomatal closure and germination. Elife. 6. doi:10.7554/eLife.23361.
